# Powering Artificial Enzymatic Cascades with Electrical Energy

**DOI:** 10.1002/anie.202001302

**Published:** 2020-04-28

**Authors:** Ammar Al‐Shameri, Marie‐Christine Petrich, Kai junge Puring, Ulf‐Peter Apfel, Bettina M. Nestl, Lars Lauterbach

**Affiliations:** ^1^ Technical University of Berlin Institute of Chemistry Strasse des 17. Juni 135 10623 Berlin Germany; ^2^ Ruhr-University Bochum Inorganic Chemistry Universitaetsstrasse 150 44780 Bochum Germany; ^3^ Fraunhofer UMSICHT Osterfelder Strasse 3 46047 Oberhausen Germany; ^4^ Universitaet Stuttgart Institute of Biochemistry and Technical Biochemistry Department of Technical Biochemistry Allmandring 31 70569 Stuttgart Germany

**Keywords:** electrochemical biocatalysis, hydrogenases, imine reductases, isotopic labeling, N-heterocycles

## Abstract

We have developed a scalable platform that employs electrolysis for an in vitro synthetic enzymatic cascade in a continuous flow reactor. Both H_2_ and O_2_ were produced by electrolysis and transferred through a gas‐permeable membrane into the flow system. The membrane enabled the separation of the electrolyte from the biocatalysts in the flow system, where H_2_ and O_2_ served as electron mediators for the biocatalysts. We demonstrate the production of methylated N‐heterocycles from diamines with up to 99 % product formation as well as excellent regioselective labeling with stable isotopes. Our platform can be applied for a broad panel of oxidoreductases to exploit electrical energy for the synthesis of fine chemicals.

## Graphical abstract

**A bioelectrochemical flow system** has been developed to power enzymatic cascades in vitro. H_2_ and O_2_ are generated by electrolysis and serve as electron mediators for oxidoreductases. Methylated N‐heterocycles were generated from diamines with up to 99 % product formation and a labeling regioselectivity of up to 99 %.

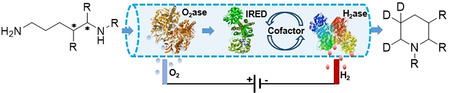

The use of electrical energy to perform biological and chemical reactions has gained extensive interest in recent years. Electro‐driven reactions offer the advantages of being clean, easy to tune, and sustainable when coupled with renewable energy sources. Biological electron transfer reactions are performed predominantly with cofactor‐dependent oxidoreductases. These represent a highly interesting and versatile class of biocatalysts for specific reduction, oxidation, and oxyfunctionalization reactions in organic synthesis.^1^ Electrical energy has been applied in various biotechnological approaches to drive whole cells or immobilized enzymes on electrodes towards cofactor recycling and production of biofuels and fine chemicals.^2^ In this context, electrochemical water splitting delivers H_2_ as an electron mediator required for redox reactions with H_2_ splitting enzymes. However, performing electro‐driven enzymatic cascades with isolated enzymes (in vitro) is still hampered by the high potentials required for water splitting, the low pH, and the high temperature generated in the process. These crucial aspects will eventually lead to denaturation of the metal‐dependent enzymes, unspecific side reactions, and the formation of undesired reactive oxygen species (ROS).2c

We therefore set out to design a novel platform to perform an electro‐driven in vitro enzymatic cascade. This one‐pot enzymatic cascade exploits the redox power of H_2_, which is generated by water splitting, in order to produce N‐heterocycles in a flow system. By utilizing a gas‐selective permeable membrane,^3^ we were able to decouple the electrochemical H_2_ generation from the enzyme transformations in one setup, thereby establishing an unhindered transfer of gases between the two liquid phases. This ensured safe H_2_ handling by avoiding the formation of explosive gas mixtures and rendered the biological system more stable. Furthermore, we established an integrated platform to monitor the relevant parameters within the system on‐line.

The bioelectrochemical system (Figure 1, top) was subsequently validated with a synthetic enzymatic cascade consisting of an immobilized oxidase, reductase, and hydrogenase to produce N‐heterocycles from diamines in a continuous process. N‐heterocycles belong to a highly important class of compounds, and are found in various natural products, biologically active structures, and medicinally relevant compounds.^4^ In previous work, we successfully showed that an NADPH‐dependent imine reductase (IRED) can be combined with an oxygen‐dependent diamine oxidase variant (PuO^E203G^) and an NADP^+^‐reducing hydrogenase in a one‐pot process for the selective synthesis of N‐heterocycles.^5^

**Figure 1:**
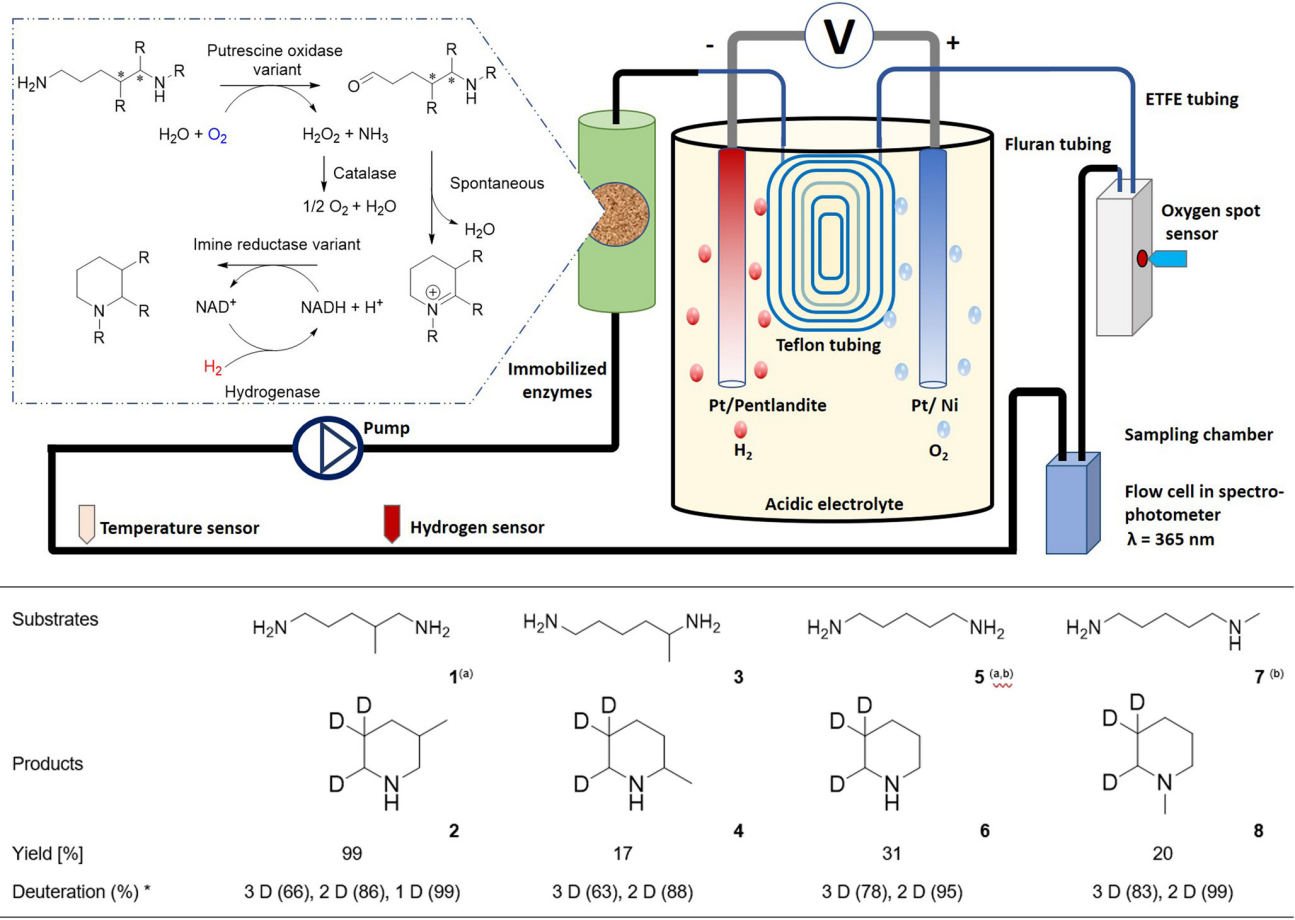
Platform of the electro‐driven in vitro enzymatic cascade for the synthesis of N‐heterocycles from diamines in a flow reactor. Both H_2_ and O_2_ are delivered by electrolysis using Pt/pentlandite as HER and Pt/Ni as the OER catalyst in an acidic electrolyte. A gas‐permeable tube transfers H_2_ and O_2_ from the electrolysis chamber (250 mL) into the flow system (16 mL). Sensors for H_2_, O_2_, and temperature and a spectrophotometer (NADH) were integrated into the flow reactor to monitor on‐line the evolution and consumption of H_2_ and O_2_ and NADH, respectively. The enzymes of the cascade (left side) were immobilized and packed into a column within the flow system. Electro‐driven cascades were performed in deuterated Tris‐HCl buffer (50 mm, pD 8.0) with 5 mm diamine substrate and 2 mm NAD^+^ cofactor in the flow reactor at 22 °C for 16 h. O_2_ and H_2_ were generated by performing electrolysis for 5 hours using Pt/Pt electrodes at 2 V and pH 2.0. Product formation (in %) was determined by GC‐FID. The regioselective labeling was confirmed by ^1^H NMR analysis (Figures S10–S18). The labeling yield was determined by comparing the signals in LC‐MS spectra (Figure S6–S9). a) Electrolysis with pentlandite/Ni electrodes at 3.5 V and pH 1.3. b) Substrate concentration 8 mm. * *x* D indicates the incorporation of *x* deuterium atoms into the product. R=methyl.

For this study, we decided to couple an NADH‐dependent IRED variant^6^ from *Myxococcus stipitatus* with the O_2_‐tolerant, NAD^+^‐reducing hydrogenase (SH) from *Ralstonia eutropha* 7 using molecular H_2_ as the reductant. We also exploited the versatility of this electro‐driven approach for 1) the synthesis of methylated N‐heterocycles and 2) the isotopic labeling of N‐heterocycles, which gave insight into the reaction mechanism of IREDs. Electrolysis was performed using a pentlandite and Ni catalysts for the hydrogen evolution reaction (HER) and the oxygen evolution reaction (OER), respectively. Pentlandite is a Ni‐ and Fe‐rich metal sulfide. It is a cheap and a sustainable alternative to platinum for HER in operations performed under conditions poisonous to other catalysts.^8^ The pentlandite/Ni system was benchmarked against the more classical Pt system. Notably, both systems were comparable in terms of efficiency with a variable dependency of H_2_ and O_2_ evolution on the pH and voltage (Figure S1–S3 in the Supporting Information). In preliminary experiments, we observed that pentlandite could produce extensive amounts of H_2_ at a low voltage of 0.6 V, leading to displacement of the trace amounts of O_2_ generated. Thus, the required amounts of O_2_ could only be generated at high voltages with Ni as OER. Moreover, the low solubility of the gases in the electrolyte negatively affected the transfer efficiency of the gases through the membrane. Thus, increasing the gas concentrations in the electrolyte by applying high voltages was crucial to enhance the transfer efficiency.^9^ Therefore, we decided to perform the electrolysis at 2 V and pH 2.0 for Pt/Pt and at 3.5 V and pH 1.3 for pentlandite/Ni.

The conversion of the model substrate 1,5‐diamino‐2‐methylpentane (**1**) into 3‐methylpiperidine (**2**) was subsequently investigated with in situ produced H_2_ and O_2_ in the enzymatic cascade. A mixture of purified enzymes was thus injected into the circulating system to start the transformation of the test substrate **1**. First results showed the successful formation of **2** with a production formation of 53 % within 16 hours (Figure S4) independent of the electrodes used. The general applicability of the flow reactor was then tested for the preparation of piperidine (**6**) and its derivatives using immobilized enzymes in the cascade reaction. While SH and catalase were immobilized on Amberlite FP54^TM^,^10^ enzyme carrier EziG^TM^ from EnginZyme^11^ was used for immobilization of PuO^E203G^ and IRED^NADH^. The specific activities per carrier ranged from 0.64 to 1 U mg^−1^ (Table S1). The immobilized enzymes were packed in a column that was integrated into the flow reactor. The evolution and consumption of O_2_/H_2_ and NADH, respectively, were monitored during the cascade using *N*‐methylcadaverine (**7**) as the model compound (Figure 2). The on‐line monitoring enabled the recording and tracking of each step of the catalysis by checking if the O_2_ and H_2_ concentrations were sufficient to start/proceed with the reaction. The monitoring also gave a comprehensive picture of whether each enzyme was functioning correctly and ensured that no parameter was limiting within the cascade. It is worth mentioning that such a monitoring system (Figure 1) had previously only been used in vivo during fermentations.

**Figure 2:**
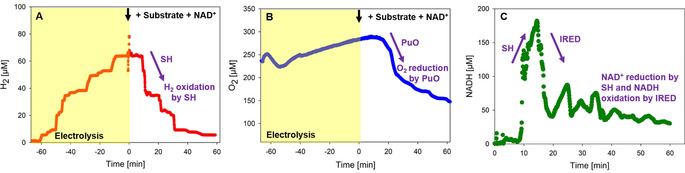
On‐line monitoring of H_2_, O_2_, and NADH during the electro‐driven biotransformation. The concentrations of H_2_ (A), O_2_ (B), and NADH (C) were followed during the first hour of the transformation of **7** into **8** as an exemplary biotransformation. The electrolysis was performed for 1 hour (yellow box) until both the H_2_ and O_2_ concentrations reached a plateau and stopped increasing. Then substrate and NAD^+^ were added (black arrows). Purple arrows indicate the activity of each enzyme: the activity of putrescine oxidase (PuO^E203G^) is indicated by the consumption of O_2_ after adding the substrate (B); the activity of imine reductase (IRED^NADH^) is shown in the consumption of NADH (C); and the activity of soluble hydrogenase (SH) is shown in the consumption of H_2_ (A) and the evolution of NADH (C). The formation of H_2_O_2_ was monitored over time; no H_2_O_2_ formation was detected. The experiment was performed as described in Figure 1.

Notably, we prepared various methylated N‐heterocycles with product formations of up to 99 %. The results obtained in the conversion of **1** and **3** were comparable to those from previous work using native IRED and NADP^+^‐reducing SH.^5^ Interestingly, in this approach, 1,5‐diaminopentane (**5**) was poorly converted. The poor conversion might be explained by the application of the IRED^NADH^ variant. The *K*_M,NADH_ value of this variant is ten times higher than the *K*_M_ value of its wild type for NADPH. The *k*_cat_ value of IRED^NADH^ is 41 % lower than that of wild‐type IRED for the reduction of the model substrate 2‐methylpyrroline.^6^ We have also observed product inhibition on IRED^NADH^ for **2** and **6** (Figure S22). Herein, a scale‐up of the reaction was easily made possible by increasing the reaction volume (up to 300 ml). We produced 69 mg of **8** and 55 mg of **2** in a 300 mL and 150 mL setup, respectively, using a threefold and twofold excess of the immobilized enzymes (Table S2). Furthermore, we produced 3 mg of *N*‐methylpyrrolidine from linear *N*‐methylputrescine (11 % yield) by using the same set of immobilized enzymes on a 150 mL scale.

The new setup was then used to test the reusability of the immobilized enzymes. The same set of immobilized enzymes could produce **6** from **5**, with 26 % of the maximum product formation retained after six cycles (Figure S5). The total turnover numbers were very high for all biocatalysts (Tables 1 and S3), demonstrating the operational stability of our system.

**Table 1:** Total turnover numbers (TTN *n*_product_/*n*_enzyme_) of each biocatalyst based on the sum of products formed after the six cycles using the same set of immobilized enzymes.

Biocatalyst	SH	IRED^NADH^	PuO^E203G^	Catalase
TTN	1.1×10^6^	1.6×10^4^	1.2×10^4^	1.6×10^8^

Finally, we studied the labeling of piperidines with stable isotopes such as deuterium in the electro‐driven cascade. The preparation of organic compounds labeled with hydrogen isotopes is of essential importance in the chemical, biological, and environmental sciences. Hence, deuterated fine chemicals are valued molecules for spectroscopic analysis, pharmaceuticals, and analytical tracing.^12^ Herein, we exploited the unique characteristic of the SH to synthesize deuterated NAD(D). SH splits H_2_ at the hydrogenase module separately from the NAD^+^ reduction at the diaphorase module. H_2_‐driven labeling can be achieved using D_2_O by providing two electrons from H_2_ splitting and D^+^ from D_2_O‐generating NAD(D).^13^ NAD(D) can then be utilized as a cofactor for various NADH‐dependent reductases such as IRED to produce deuterated compounds (Scheme 1).

**Scheme 1:**
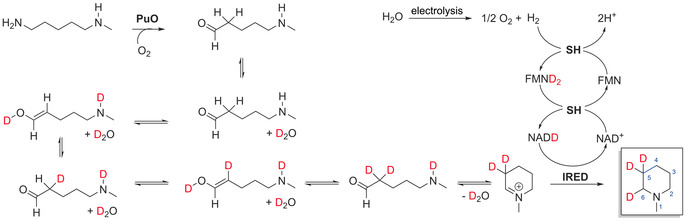
Proposed mechanism of isotope labeling by several rounds of keto–enol tautomerization (C5) and SH activity (C6) with the model compound **7**. The iminium ion is formed during cyclization. The SH enzyme catalyzes the combined H_2_ oxidation and NAD^+^ reduction activity. Electrons are transferred via the prosthetic Fe‐S clusters and flavins to the NAD binding site. The FMN at the NAD^+^‐binding site is converted by two electrons and two deuterium ions from water into FMND_2_. A deuteride transfer from FMND_2_ to NAD^+^ generates NAD(D).^14^ PuO: putrescine oxidase variant E203G; IRED: NADH‐dependent imine reductase; SH: NAD^+^‐reducing hydrogenase.

The labeling was performed with D_2_O in the biotransformation and H_2_O in the electrolysis units. We produced various labeled piperidine derivatives from diamines with up to 99 % of deuterium labeling. Only a small fraction of the products was partially labeled (Figure 1). This inhomogeneity can be explained by the presence of water traces in D_2_O (purity 98 %), protons released from H_2_ splitting (ca. 1 %), residual water in the gases, remaining H_2_O from immobilized enzymes, and the natural proton abundance of NAD^+^. We expected to observe labeling with a single D atom at the C6 carbon atom of the imine. Interestingly, we observed further labeling with two D atoms at C5 (for the MS and NMR spectra, see Figures S6–S18). We hypothesize that the deuteration at C5 might have been caused either by a possible keto–enol tautomerization, followed by the oxidation of the substrate by PuOE^203G^, or an imine–enamine tautomerization with subsequent spontaneous cyclization of the aminoaldehyde intermediate. To examine this hypothesis, we conducted the isotopic labeling using imines (2‐methylpyrroline and 3,4‐dihydroisoquinoline) and IRED^NADH^. Here, the labeling is facilitated only by the activity of SH. Mass and NMR spectra revealed a 85 % labeling corresponding to the C6 carbon atom of the imine (Figures S19–S21) without any labeling on further atoms. In addition, we used formate dehydrogenase (FDH) for cofactor regeneration in control experiments. No labeling was observed when FDH was used. This finding indicates that the deuteration at C5 was merely caused by the keto–enol tautomerization (Scheme 1).

We determined that only about 4 % of the electrical energy was used for product formation (see Chapter S2.11 in the Supporting Information). While this value seems very low, this finding can be rationalized by the low solubility of the gases in water, reduction of O_2_ at the HER, heat production by overpotentials, inefficient gas transfer through the membrane, and product inhibition of IRED^NADH^. Our system can be further optimized by using suitable gas exchange membranes, sophisticated electrodes, and further engineered biocatalysts with improved features and broader substrate scopes.

In conclusion, we have designed a scalable platform to power enzymatic cascades with electricity. We furthermore demonstrated the potential of the novel design by producing various piperidine derivates from diamines. We extended the applicability of the system towards performing regioselective isotopic labeling and provided useful insight into imine reduction by IREDs. This platform represents an important advance in the field of biocatalytic synthesis, and it can be expanded for powering various cofactor‐dependent oxidoreductases.

## Conflict of interest

The authors declare no conflict of interest.

## Supporting information

As a service to our authors and readers, this journal provides supporting information supplied by the authors. Such materials are peer reviewed and may be re‐organized for online delivery, but are not copy‐edited or typeset. Technical support issues arising from supporting information (other than missing files) should be addressed to the authors.

Supplementary
