# Supplementary material for: Powering Artificial Enzymatic Cascades with Electrical Energy
Source: Angew Chem Int Ed Engl. 2020 Apr 28;59(27):10929–33. doi: 10.1002/anie.202001302 (PMC7318245; doi:10.1002/anie.202001302)
Supplement: Supplementary file 1 — Supplementary [file ANIE-59-10929-s001.pdf]

Angewandte Chemie
Eine Zeitschrift der Gesellschaft Deutscher Chemiker

Supporting Information

Powering Artificial Enzymatic Cascades with Electrical Energy

*Ammar Al-Shameri, Marie-Christine Petrich, Kai junge Puring, Ulf-Peter Apfel, Bettina M. Nestl, and Lars Lauterbach**

anie_202001302_sm_miscellaneous_information.pdf

Table of Contents

1. Experimental Procedures 1
1.1. Chemicals and materials 1
1.2. Enzyme purification and enzyme immobilization 1
1.3. Construction of pentlandite electrode 1
1.4. Mini flow reactor 2
1.5. Electro-driven biocatalysis 3
1.6. Deuteration 3
1.7. H_2O_2 detection 3
1.8. Product isolation 4
1.9. Analysis 4
1.9.1. GC 4
1.9.2. ESI-LC-MS 4
1.9.3. NMR 4
2. Results and Discussion 5
2.1. The evolution of the gases by electrolysis 5
2.2. Electro-driven biotransformation with purified enzymes 7
2.3. Enzyme Immobilization 8
2.4. The reusability of the immobilized enzymes 8
2.5. Scaling up 9
2.6 MS spectra 10
2.7 NMR spectra 14
2.8 Experiments with IRED 21
2.9 Product inhibition of IRED 25
2.10 Monitoring product formation 25
2.11 Calculation for total energy conversion yield 26
3 References 27

1. Experimental Procedures

1.1. Chemicals and materials

Except otherwise noted, all solvents, buffer components and chemicals were obtained from SigmaAldrich, Merck and Fluka (Steinheim, Germany), Carl Roth GmbH (Karlsruhe, Germany) and Alfa Aesar (Karlsruhe, Germany). Putrescine and 1,5-diaminopentane were obtained from VWR (Darmstadt, Germany). 1,5-diamino-2-methyl-pentane was purchased from Sigma Aldrich (Steinheim, Germany) as well as the standards pyrrolidine and (*S*)-2-methylpiperidine. Piperidine, 3-methylpiperidine and 2-methylpiperidine were obtained from Merck (Steinheim,Germany). 1,5-Diaminohexane was obtained from FCH Group. *N*-methyl 1,5-diamino-pentane was purchased from Chemspace (Riga, Latvia). Oxygen adhesive spot sensor was purchased from PreSens (Regensburg, Germany. H_2 sensor was obtained from Unisense (Aarhus, Denmark). Teflon AF 2400 tube Ø 0.81 x 0.62 mm x 90 cm was purchased from biogeneral (San Diego, USA). Mini flow cuvette was obtained from Hellma analytics (Müllheim, Germany). NanoPhotometer from Implen (Munich, Germany) was used for spectrophotometric analysis. Titnium electrodes Ø 8 mm x 100 mm coated with 2,5 µm Platinum was purchased from Polymet (Lüneburg, Germany). Amberlite^{TM} FPA54 was purchased (Rohm & Haas) whereas EziG^{TM} carriers were kindly provided by EnginZyme (Stockholm, Sweden). Fluran®, F-5500-A pipe Ø 3.2 mm was purchased form VWR (Darmstadt, Germany).

1.2. Enzyme purification and enzyme immobilization

The production and the purification of putrescine oxidase from *Rhodococcus erythropolis* (PuO^{E203G}) and imine reductase from *Myxococcus stipitatus* (IRED^{NADH}) were performed as described in Al-Shameri *et al.* [1] NAD^+-reducing hydrogenase (SH) was produced and purified as described in Lauterbach *et al*.[2] Catalase was purchased from Merck Fluka (Steinheim, Germany). SH and catalase were covalently immobilized on Amberlite as described in Herr *et al* [3] using a ratio of (1:1000) of enzyme to carrier. PuO^{E203G} and IRED^{NADH} were immobilized on EziGI according to manufacturer´s protocol. 1 g cell mass of each PuO^{E203G} and IRED^{NADH} were dissolved in buffer A (20 mM phosphate buffer containing 500 mM NaCl and 20 mM imidazole, pH 7.5). Cells were disrupted by passing them twice through French press. Cell debris was removed by centrifugation (30 min, 4 °C, 9000 X g). Cell free extracts were incubated with 0.5 g EziGI^{TM} for 1 hour at 4 °C on an orbital shaker. EziGI^{TM} was removed by sedimentation and washed twice with 50 mM Tris-HCl pH 8. The EziGI^{TM} loaded with enzyme was stored at 4°C.

1.3. Construction of pentlandite electrode

The pentlandite electrodes were manufactured using a facile dry-pressing method. A mixture of carbon black (ENSACO 250G, Imery´s Graphite & Carbon), pentlandite (10 wt.% with respect to carbon black) and PTFE (Dyneon^{TM} PTFE Powder TF 1750, 3M, 15 wt.% with respect to carbon black) were mixed for 1 min in an IKA M-20 universal blade mill for 1 min. The powdered mixture (2 g) was doctor-bladed on the MPL side of a CeTech W1S1009 Carbon Cloth (+MPL) gas diffusion layer using a 90x70x3 mm stainless steel mask. The mixture was then pressed on the gas diffusion layer the mask at 260 °C and 3.6 kN cm^{-2} for

10 min without the mask. For electrochemical experiments, smaller electrodes could be cut from the large sheet using scissors.

1.4. Mini flow reactor

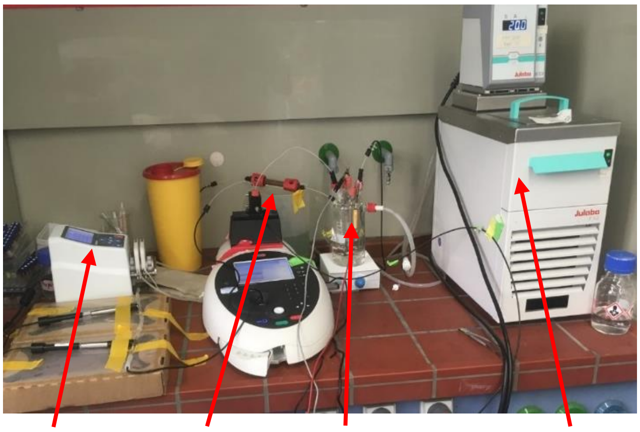

Pump Enzyme column Electrolysis Thermo controller

H_2 sensor Temperature sensor Photometer for NADH monitoring

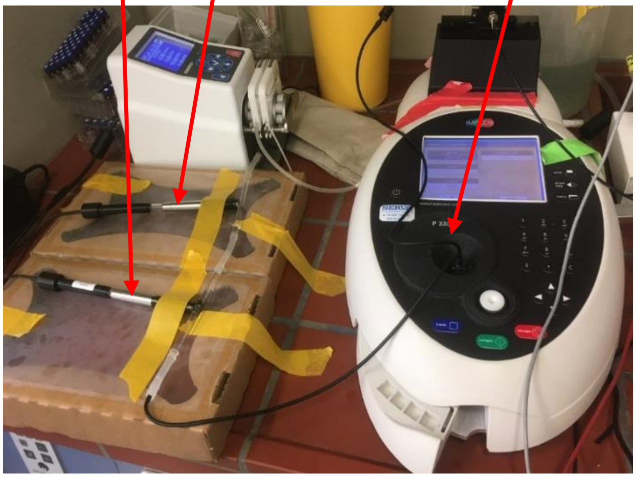

Cooling Electrod O$_2$ spot Enzyme

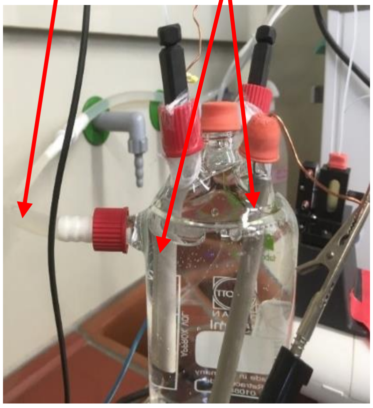

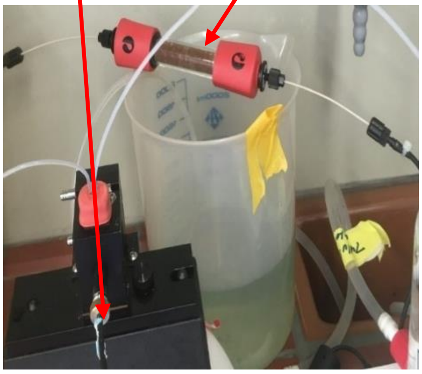

- The electrolysis is taking place in the electrolysis chamber (250 ml); both electrodes (pentalandlite/Pt and Ni/Pt) are immersed in electrolyte (pH 1, 10 mM H_2SO_4). The electrolysis unit was filled with electrolyte completely to avoid the formation of gas phase.
- Pt electrodes were titanium electrodes Ø 8 mm x 100 mm coated with 2,5 µm platinum. pentalandlite/Ni: Ni net (9 X 1.5 cm) and pentalandlite (8 X 1.7 cm).
- Teflon AF 2400 tube Ø 0.81 x 0.62 mm x 90 cm is immersed is the electrolyte, and connected to Fluran®, F-5500-A pipe Ø 3.2 mm. Which is connected to a pump.
- The reaction buffer is pumped through the pipe and Teflon tube. H_2 and O_2 produced by electrolysis are transferred through the Teflon tube to the reaction buffer.
- Furthermore, the flow system includes: a flow cuvette, located in spectrophotometer to measure the concentration/consumption of NADH at 365 nm, a cuvette with spot sensor (PreSens) stuck to a transparent cuvette to measure O_2, and a sensor to measure H_2 and temperature.
- For the setup with immobilized enzymes, the flow system included additionally a column packed with enzymes carriers.
- The electrolysis chamber was cooled with water, which temperature is controlled by a thermostat 20 °C. The electrolyte is constantly mixed using a magnetic stirrer to ensure a homogenous distribution of the gases.

1.5. Electro-driven biocatalysis

- The flow system (16 ml) was filled with buffer 50 mM Tris-HCl pH 8 at room temperature.
- Flow rate was set at and at 80 ml/min but switched to 1 ml/min when the column with immobilized enzymes was inserted to avoid the overpressure.
- Electrolysis was performed for 1 hours at max 2 V pH 2 is case of platinum electrodes and max 3.5 V pH 1.3.
- NAD^+ was added in final concentration of 2 mM.
- Substrate was added in final concentration of 5-10 mM.
- The concentrations of temperature, O_2, H_2 and NADH were monitored throughout the whole reaction.
- Samples were taken at different time points and extracted and analyzed via GC-FID.

1.6. Deuteration

The cascade was performed similar as mentioned above. Tris-HCl buffer was adjusted at pH 7.5, the water was then removed by lyophilisation. The remained salts were dissolved in D_2O, then procedure was repeated. and the final concentration was set at 50 mM by dissolving the remade salts in D_2O. The flow system including the column of enzymes was equilibrated with 50 ml deuterated buffer before starting the reaction.

1.7. H_2O_2 detection

The H_2O_2 concentration in the biotransformation was monitored over time using H_2O_2 detection colometric strips MQuant® from Merck.

1.8. Product isolation

For final product isolation, 1.5 M NaOH final concentration was added tot whole reaction and extracted with 70% *v/v* MTBE five times. MTBE was removed using rotary evaporator (40 °C, 0.5 bar). At the end, the pressure was decreased to 0.2 bar to remove the residual traces of MTBE

1.9. Analysis

1.9.1. GC

5 M NaOH was added to the samples vol. (1:1). Samples were then acetylated using acetic anhydride after adding 2-methyl pyrrolidine as internal standard and extracted with methyl-*tert*-butyl ether. 4 µL of the extracted sample was injected into GC-FID.

GC-FID analyses of performed biotransformations were carried out using a Shimadzu GC-2010 equipped with an AOC-20i auto injector. The injector temperature was 250°C and compounds were detected *via* flame ionization detector (FID) at 330°C or 255°C. Identification of compounds was performed by comparing the retention time of the produced compounds with commercially available reference materials, piperidine, 3-methylpiperidine, and 2-methylpiperidine. A DB-5 capillary column (Restek 5 MS, length 30 m × 0.25 mm × 0.25 µm) was used with H2 as carrier gas (51.3 mL/min, linear velocity 30 cm/s) and samples were injected in split mode (1:50). The temperature program was as following: 1 min at 70°C, 15°C/min to 90°C, 20°C/min to 185°C, hold 1 min, 25°C/min to 300°C and hold for 3 min.

1.9.2. ESI-LC-MS

HPLC-HR-ESI-(+)-Orbitrap-MS mass spectra were recorded using a LTQ-Orbitrap XL (Thermo Scientific, Bremen, Germany) coupled to an Agilent 1260 HPLC system (Agilent Technologies, Waldbronn, Germany). Samples were filtered using 0.2µm filter and injected into HPLC at room temperature. The HPLC measuring conditions are as follows:
Column: Grom-Sil-120-ODS-4-HE (Grace, length 50mm, ID 2mm, 3µm)
Gradient flow: Eluent 1: H_2O + 0.1% *v/v* HCOOH and Eluent 2: MeCN + 0.1% *v/v* HCOOH. 0 - 10 min: Eluent 2: from 20% to 100%, 10 - 13 min: Eluent 2: hold 100 % and 13 - 18 min: Eluent 2: hold 20 %.
Flow rate: 0.3 mL / min.
DAD spectrum scan range 205 nm – 850 nm ; Step 2 nm. UV/Vis channel: channel A – 215 nm; 4nm bandwidth. channel B – 280 nm; 40nm bandwidth. channel C – 350 nm; 100nm bandwidth.
Raw data were processed with Mass++, mass spectra were plotted with SigmaPlot 12.3.

1.9.3. NMR

The ^{1}D- and ^{2}D-NMR spectra were recorded on a Bruker Avance III 700 spectrometer (Bruker, Karlsruhe, Germany) Sample was dissolved in 600 µl D_2O for NMR experiments. For the calibration, the residual solvent peaks of D_2O (4.50 p.p.m. (^{1}H). All experiments were performed at T = 298 K. The spectra plots were generated using TopSpin3.5pl7.

2. Results and Discussion

2.1. The evolution of the gases by electrolysis

The evolution of the H_2 and O_2 was tested at different electrolyte pH 1.3 (50 mM H_2So_4) , 2.7 (10 mM H_2So_4) and 7 (50 mM Tris HCl). Prior to each measurement, the system and electrolyte were flushed thoroughly with N_2. The electrolysis was performed at two different voltages 2.5 V and 3.5 V for 40 minutes, both O_2 and H_2 were monitored. Figure 1S and 3S shows the evolution of the O_2 and H_2, respectively with pentlandite /Ni electrodes. The figures indicate that the combination of pH 1.3 with 3.5 V was the optimal pH and voltage to deliver the maximum H_2 and O_2 to the system. Performing the electrolysis at pH 7 lead surprisingly to high rapid evolution of H_2 and resulted in destroying the Ni electrode. The pH changed to 12 and the electrolyte turn green. Indicating that, at this pH and voltage Ni is oxidized into $NiOH_2$ instead of O_2. Figure 2S shows the evolution of O_2 at 1.5 and 2 V and pH 2 with Pt/Pt electrodes. The evolution of O_2 was at least five times higher with 2V.

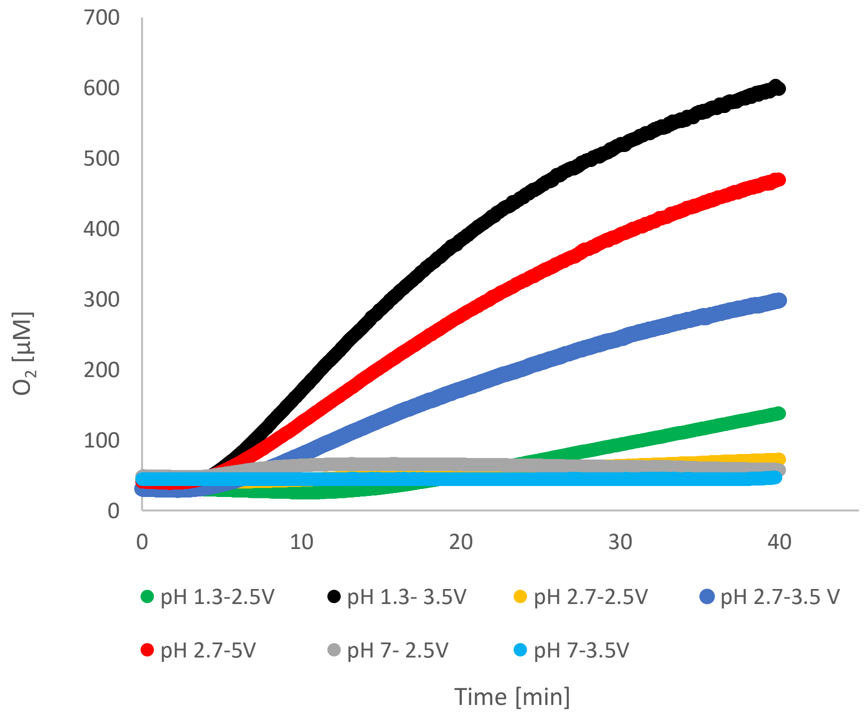

Figure 1S: The evolution of O_2 overtime at different pH (1.3, 2.7 and 7) and voltage (2.5, 3.5 and 5 V) using pentlandite /Ni electrodes.

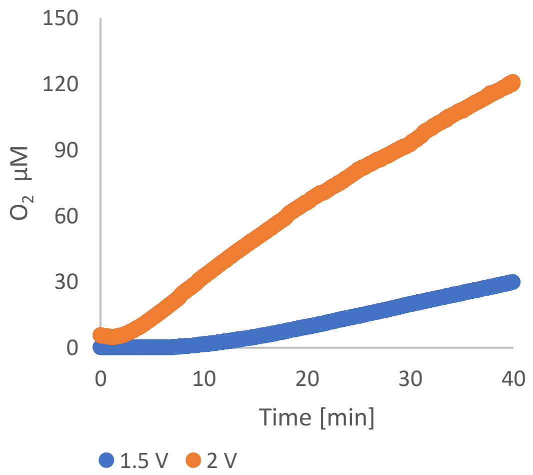

Figure 2S: The evolution of O_2 overtime at two different voltages (1.5 and 2 V) and pH 2 using Pt/Pt electrodes

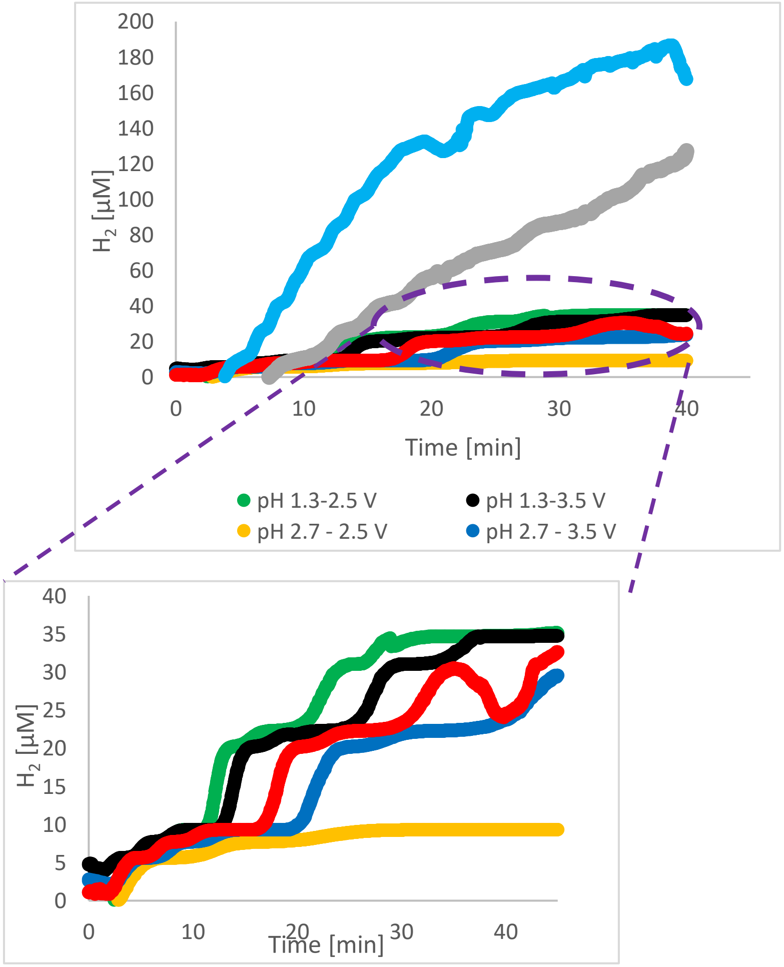

Figure 3S: The evolution of H_2 over time at different pH (1.3, 2.7 and 7) and voltage (2.5, 3.5 and 5 V) using pentlandite /Ni electrodes

2.2. Electro-driven biotransformation with purified enzymes

The conversion of 1,4-methylcadaverine into 3-methylpiperidine was tested in the flow system. The electro-driven biotransformation was performed as described in 1.5, using Pt/Pt electrodes at 2 V, pH 2 and 10 mM substrate. A mixture of purified enzymes: (PuO^{E203G} (1 µM), IRED^{NADH} (1 µM), SH (0.5 U) and catalase (1000 U)) was injected into the flow to initiate the biotransformation.

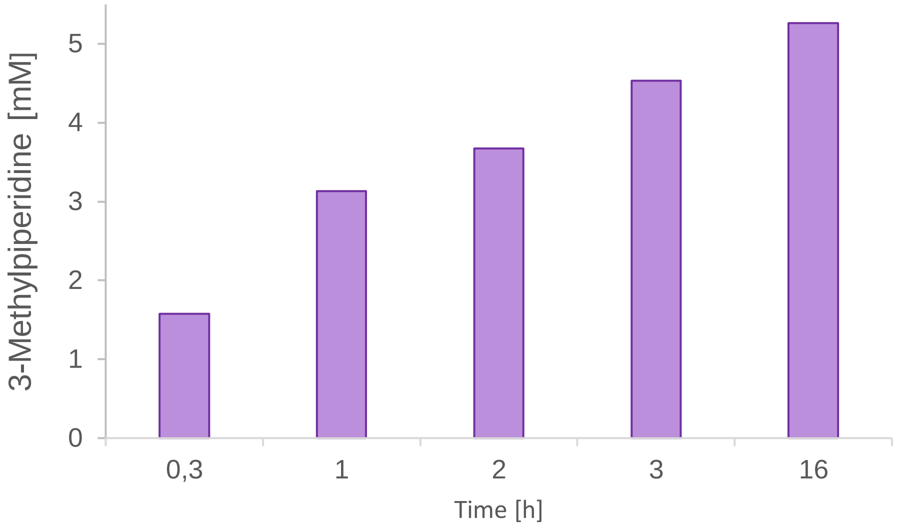

Figure 4S: The electro-driven production of 3-methylpiperidine form 1,4-methylcadaverine overtime with purified enzymes.

2.3. Enzyme immobilization

Both PuO^{E203G} and IRED^{NADH} were immobilized on EziGI™. SH and catalase covalently on Amberlite™ FPA54. IRED activity was determined by measuring the oxidation of NADH at 365 nm towards 2-methylpyrrolidine as substrate. PuO^{E203G} activity towards cadaverine was determined by measuring the oxidation of ABTS at 405 nm by horseradish peroxidase and H_2O_2. SH activity was determined by measuring the reduction of NAD^+ at 365 nm with H_2 as the reductant. Catalase activity towards H_2O_2 as substrate was determined by measuring the evolution of oxygen by the PreSens spot sensor. All activities were determined in Tris-HCl pH 7.5 at 21 °C.

Table 1S: The immobilization yield of the enzymes on the corresponded carriers

Enzyme	Specific activity [U/mg]	Activity per carrier [U/g]	Enzyme immobilized per carrier [mg/g]
IRED	0.68	1	1.6
PuO	0.5	0.64	1.3
SH	43.5	1	0.023
Catalase	5600	1	0.00018

2.4. The reusability of the immobilized enzymes

The reusability of the immobilized enzymes (400 mg of EziGI-PuO^{E203G} and 300 mg of EziGI-IRED^{NADH}, 1 g of Amberlite-SH and 1 g Amberlite- catalase) in the flow reactor was tested. The conversion of cadaverine into piperidine was analysed after multiple runs. Here, the cascade was performed as with pentalandlite/Ni electrodes for 1 h at 3.5 V and pH 2.7. 2 mM of NAD^+ and 8 mM substrate were added. Each reaction was either performed overnight or for 5 hours. After the reaction was finished, the system was washed out and equilibrated with a new buffer and a new reaction was started. Figure 5S shows the production yield of each reaction. After the sixth reaction, the yield decreased by nearly fivefold. After six runs with same immobilized enzymes nearly 20% of the product formation remained.

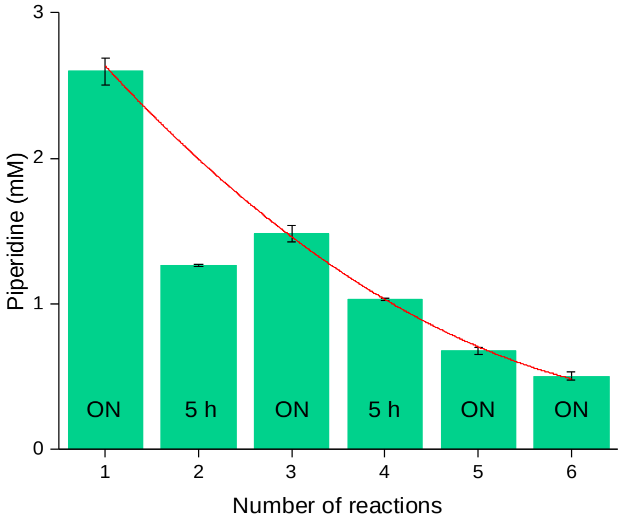

Figure 5S: The reusability of the immobilized enzymes in the production of piperidine from cadaverine.

2.5. Scaling up

The electro driven enzymatic cascade was scaled up by integrating an additional reservoir into the flow system. The electrolysis was performed with Pt/Pt system 2V. The 2-fold and 3-fold excess amounts of immobilized enzymes were used for 150 mL 300 mL.

Table 2S: Scaling up of the electro driven enzymatic cascade

Substrate	Product	Isolated yield [mg]	Scale
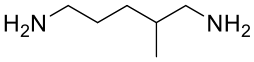 **1**	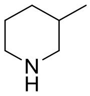 **2**	55	150 mL for 16 hours 2 g Amberlite-SH, 2 g Amberlite-catalase, 1 g EziG -IRED^{NADH} and 1 g EziG -PuO^{E203G}. 110 mg substrate
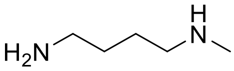 **9**	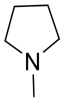 **10**	3	2nd run of same setup with 30 mg substrate
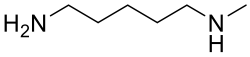 **7**	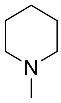 **8**	69	300 mL for 36 hours 3 g Amberlite-SH, 3 g Amberlite-catalase, 3 g EziG -IRED^{NADH} and EziG -PuO^{E203G}. 310 mg substrate

Table 3S. Total turnover numbers (TTN $n_{product}/n_{enzyme}$) of each biocatalyst in the biotransformation of **7** into **8** in 300 mL scale

biocatalyst	SH	IRED^{NADH}	PuO^{E203G}	Catalase*
TTN	7.3 x10^{5}	4.2 x10^{4}	6 x10^{4}	3.4 x10^{7}

*new catalase charge with 12637 U/mg activity. 16.6 nmol IRED^{NADH}, 11.7 nmol, PuO^{E203G} , 0.003 nmol catalase, 1 nmol SH

2.6 MS spectra

The following graphs (Figure 6S-9S) show the MS spectra of the different biotransformations conducted in D_2O. MS spectra of commercial standards were also taken as control. The spectra were plotted using Sigmaplot.

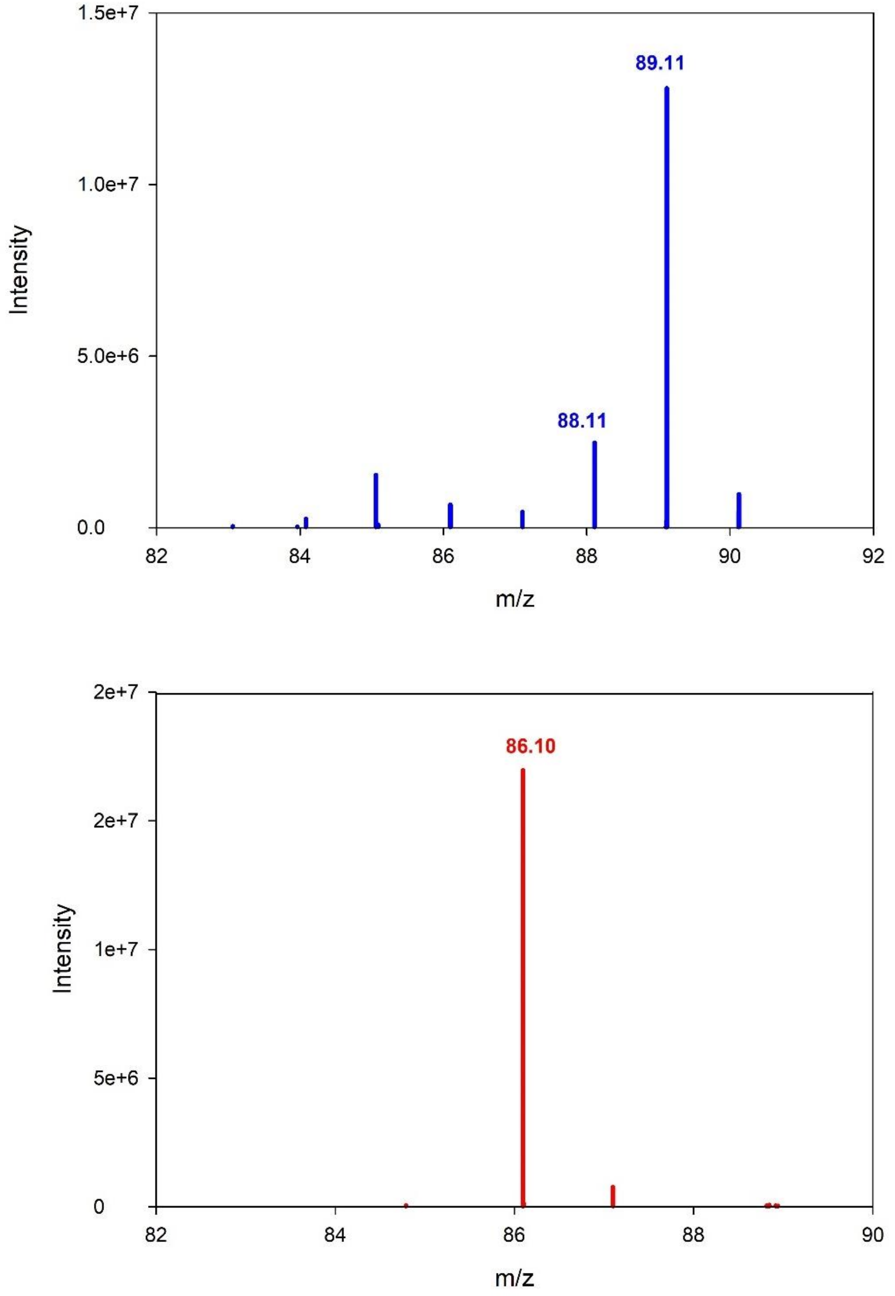

Figure 6S: Mass spectra of commercially available piperidine (below), *m/z* = 86.10 and piperidine produced from the conversion of cadaverine in the presence of D_2O (above).

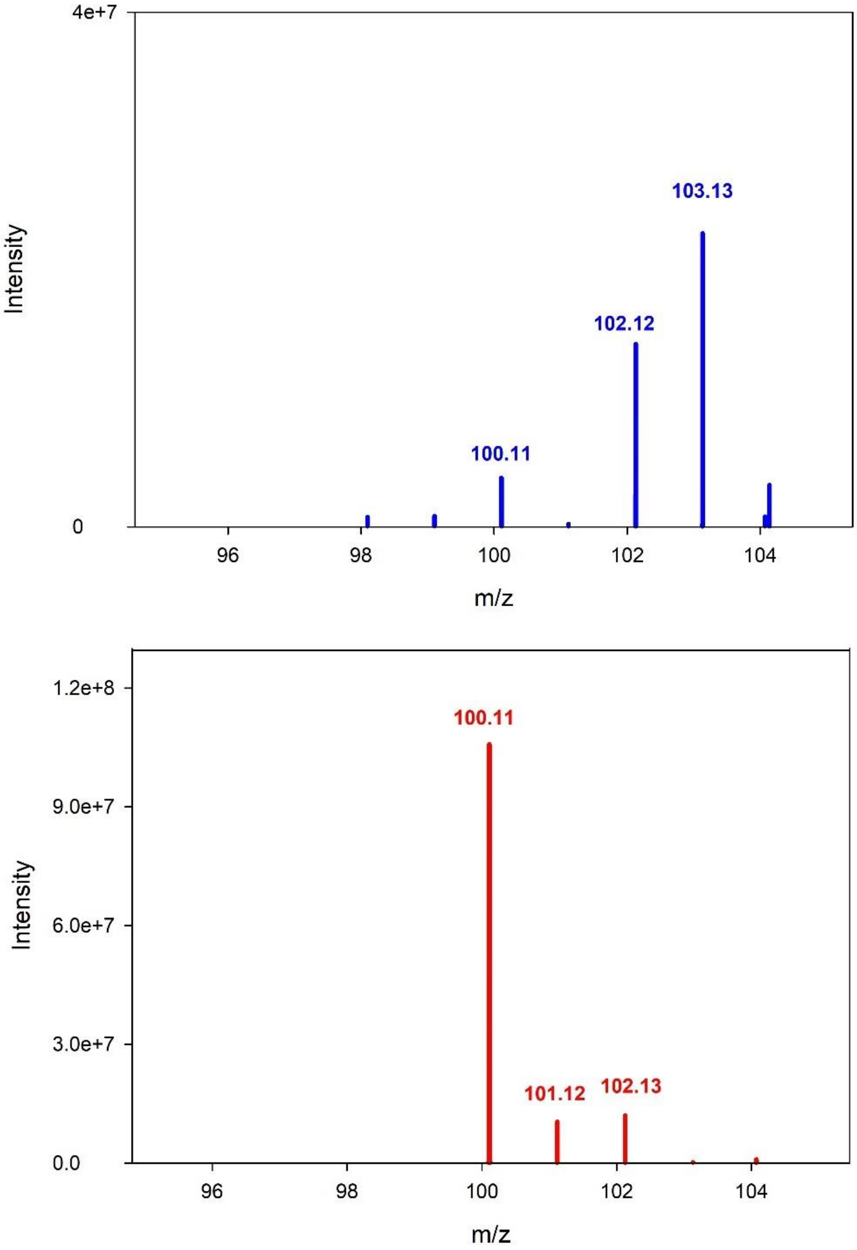

Figure 7S: Mass spectra of commercially available 2-methylpiperidine (below), *m/z* = 100.11 and 2-methylpiperidine produced from the conversion of 1,5-diaminohexane in the presence of D_2O (above).

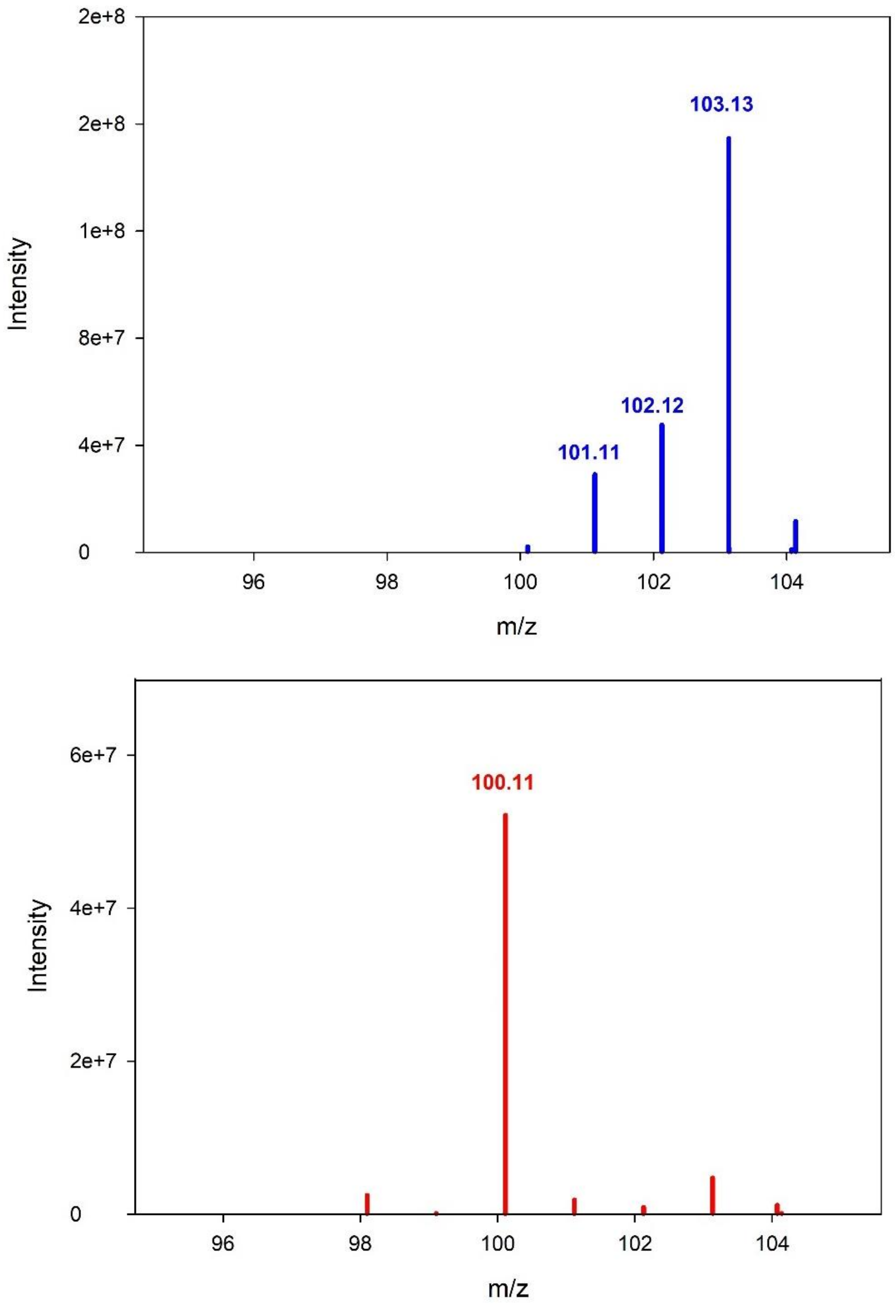

Figure 8S: Mass spectra of commercially available 3-methylpiperidine (below), *m/z* = 100.11 and 3-methylpiperidine produced from the conversion of 4-methyl-1,5-diaminopentane in the presence of D_2O (above).

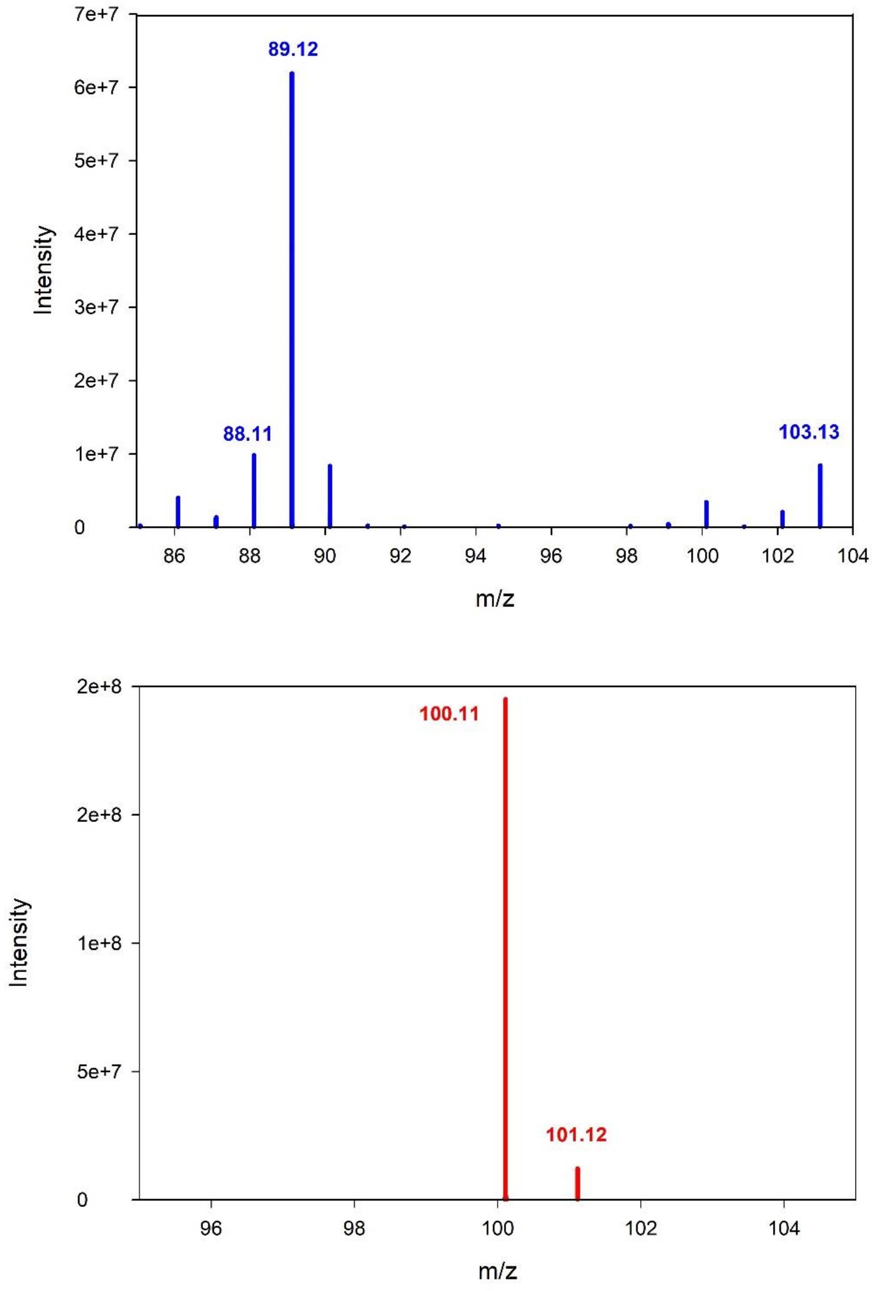

Figure 9S: Mass spectra of commercially available *N*-methylpiperidine (below), *m/z* = 100.11 and *N*-methylpiperidine produced from the conversion of *N*-methyl-1,5-diaminopentane in the presence of D_2O (above).The fragmentation of the product into deuterated piperidine is seen in sample with D_2O.

2.7 NMR spectra

The following graph (Figures 10S-13S) shows the NMR spectra of 3-methylpiperidine produced from the biotransformation of 4-methyl-1,5-diaminopentane in D_2O. NMR spectra of commercial standards were also taken as control. 2D-NMR were performed to determine the coupling of H atoms.

^1H NMR of 3-methlypiperidine in D_2O

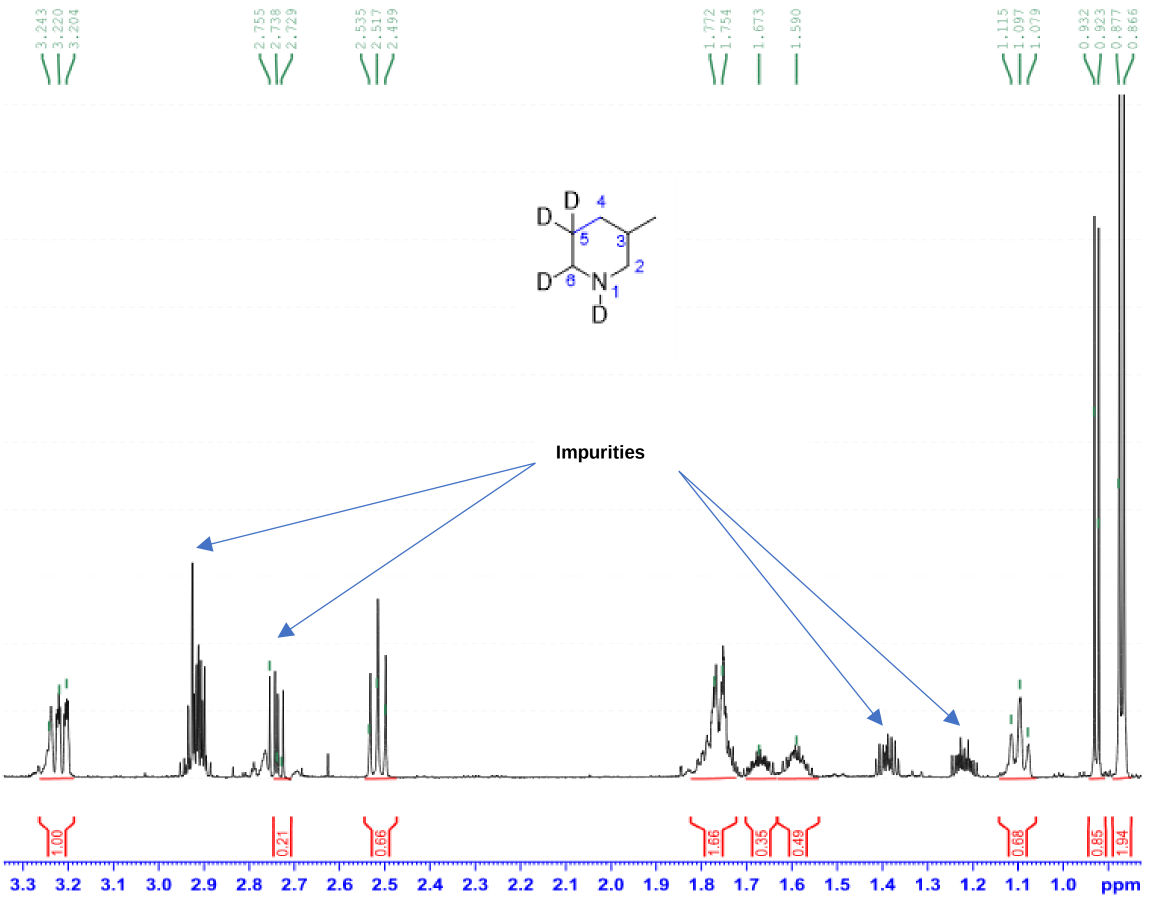

Figure 10S: ^1H NMR of 3-methlypiperidine in D_2O

^1H NMR (500 MHz, D_2O) δ (ppm): 0.87 and 0.92 (methyl group both enantiomers, d, J= 6.6 Hz), 1.097 (4a-H, dd, J = 12.5, 11.7 Hz), 1.59 (3-H, m), 1.76 (4e-H, dm, J = 13.6 Hz), 2.5 (2a-H, t, J = 12.3, 11.7), 3.2 (6e-H, singlet), 3.23 (2e-H, d, J = 12.4 Hz).

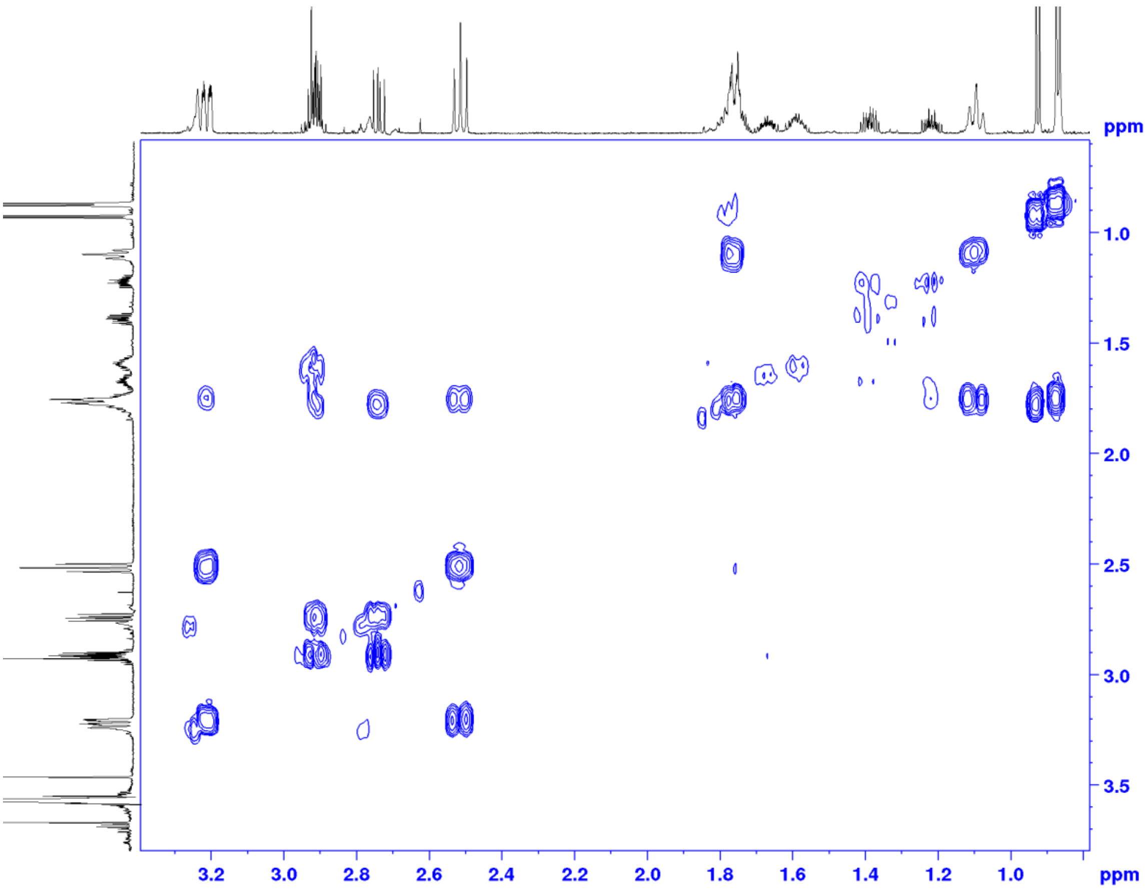

Figure 11S: ^{1}H, ^{1}H COSY of 3-methlypiperidine in D_2O.

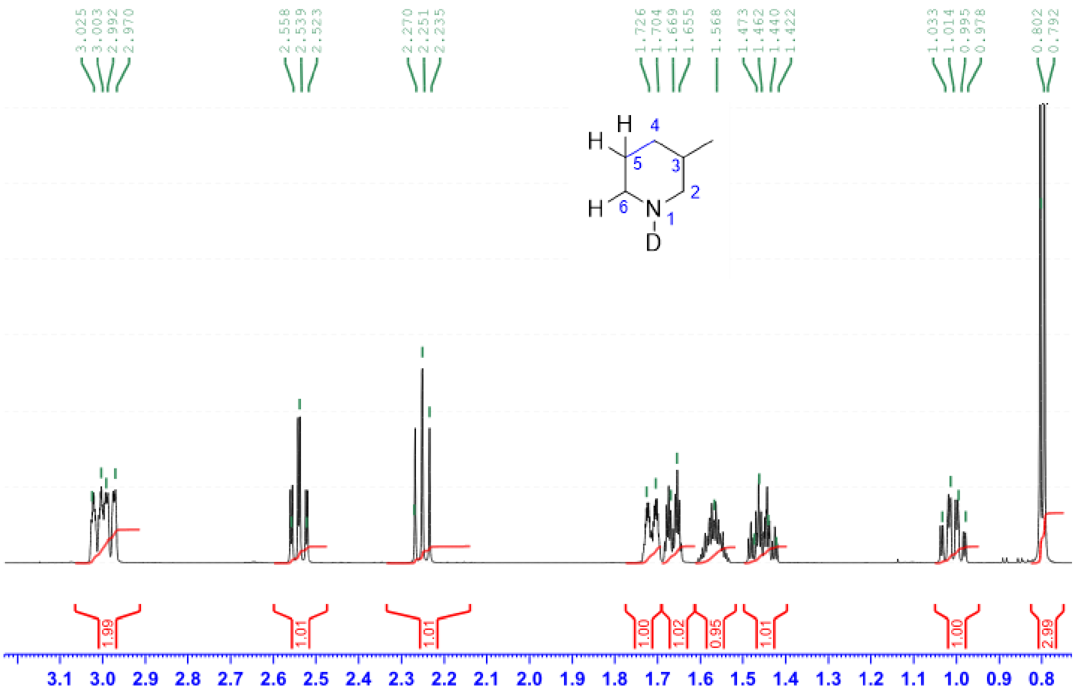

Figure 12S: ^{1}H NMR of 3-methlypiperidine standard

^1H NMR (500 MHz, D_2O) δ (ppm): 0.8 (methyl, d, *J* = 6.65 Hz), 1 (4a-H, dddd, *J* = 12.5, 12.5, 10.9, 3.9 Hz), 1.45 (5a-H, qt, *J* = 12.5, 4 Hz), 1.57 (3-H, m), 1.65 (5e-H, dm, *J* = 13.8 Hz), 1.76 (4e-H, dm, *J* = 13.6), 2.25 (2a-H, t, *J* = 12.3, 11.7 Hz), 2.53 (6a-H, td, *J* = 12.3, 3.3 Hz), 2.97 (6e-H, d, *J* = 12.3), 3.0 (2e-H, *J* = 12.4 Hz)

The slight shifts observed between the standard and the samples is due the buffer and NAD^+ presented, since samples were directly measured with NMR without extraction. When the standards were analysed using the same buffer with NAD^+ no shifts were observed. Tri-doublet at 2.5 ppm was nearly vanished indicating that 6a-H was replaced by deuterium. Both Hs at atom 5 were almost completely replaced by deuterium, because qt of 5a-H at 1.45 ppm was vanished and 5e-H at 1.65 ppm became very weak. The coupling of 6e-H at 2.97 ppm became singlet indicating that 6a was by deuterium. Moreover, the dddd of 4a-H became triplet indicating that atom 5 was replaced.

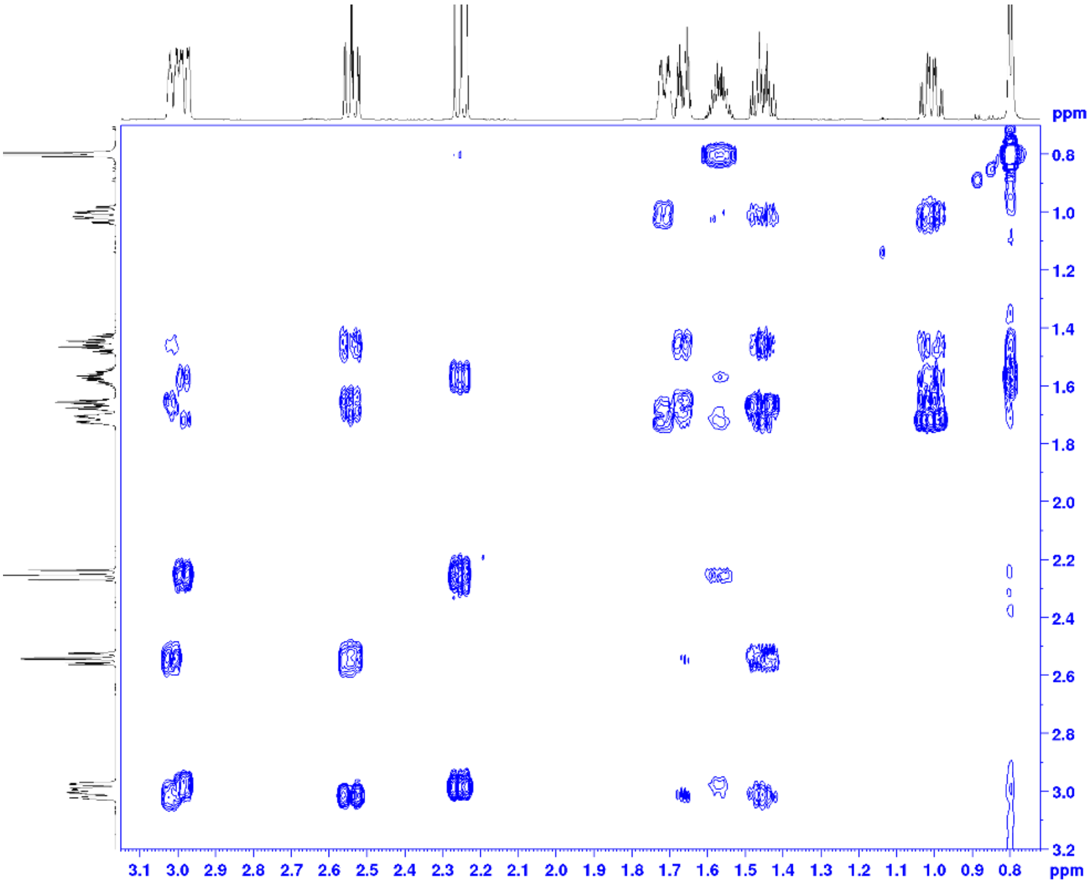

Figure 13S (A): ^1H,^1H COSY of 3-methlypiperidine (commercial standard).

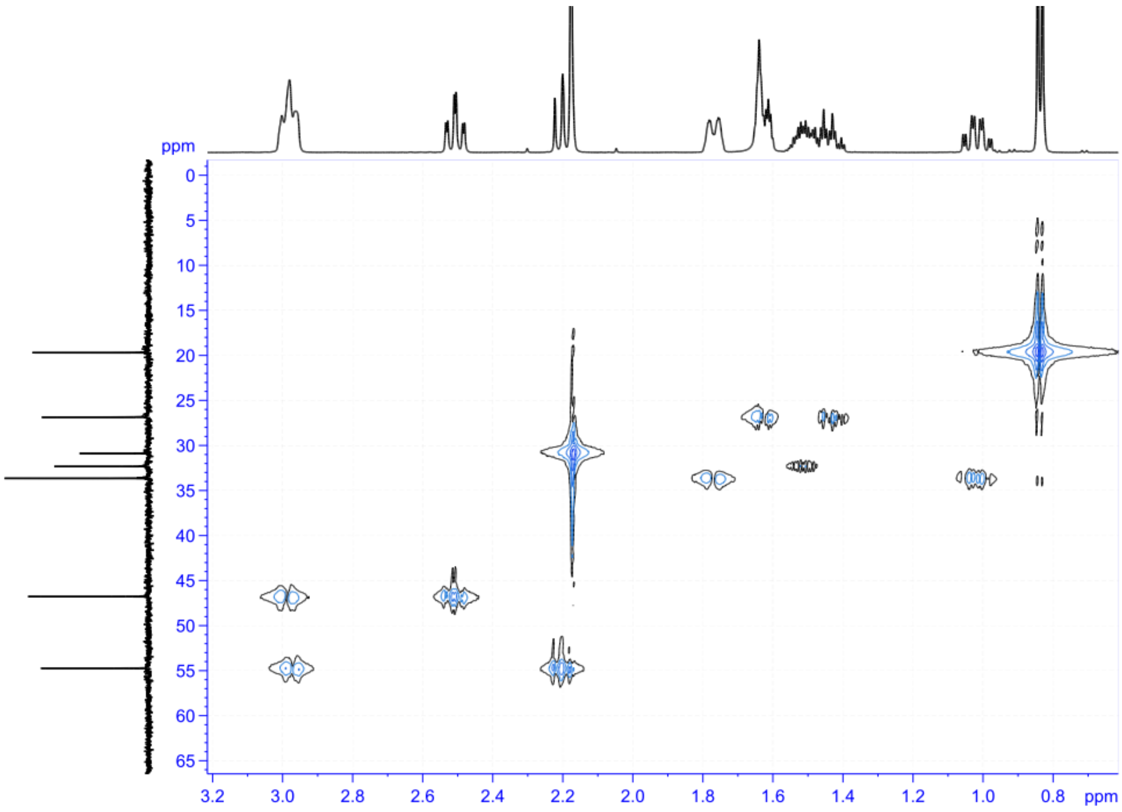

Figure 13S (B): $^{1}H,^{13}C$ HMQC of 3-methlypiperidine (commercial standard).

The following graph (Figures 14S-17S) shows the NMR spectra of piperidine produced from the biotransformation of cadaverine in D_2O. NMR spectra of commercial standards were also taken as control. 2D NMR were performed to determine the coupling of H atoms. Figure 18S shows the NMR spectra of *N*-methylpiperidine produced from the biotransformation of *N*-methyl cadaverine in D_2O.

Piperidine in D_2O

^{1}H NMR (500 MHz, D_2O) δ (ppm): 1.65 (4-H, t, J = 5.8, 5.3 Hz), 1.78 (3-H, q, J = 5.5 Hz), 3.14 (6-H, singlet), 3.16 (2-H, t, J = 5.5 Hz), traces of t: 7.7 Hz for 6H at 3.25.

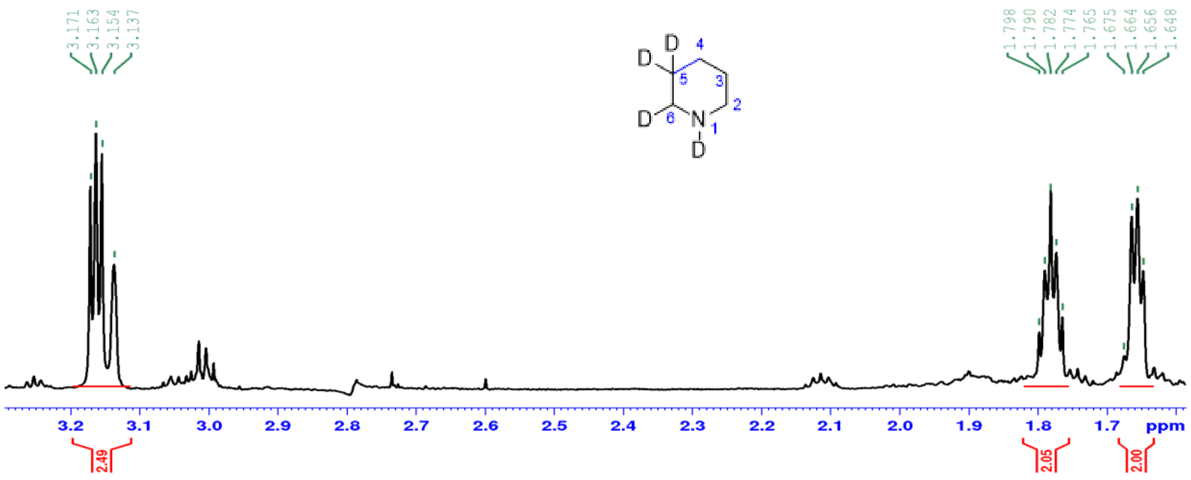

Figure 14S: ^{1}H NMR of piperidine in D_2O

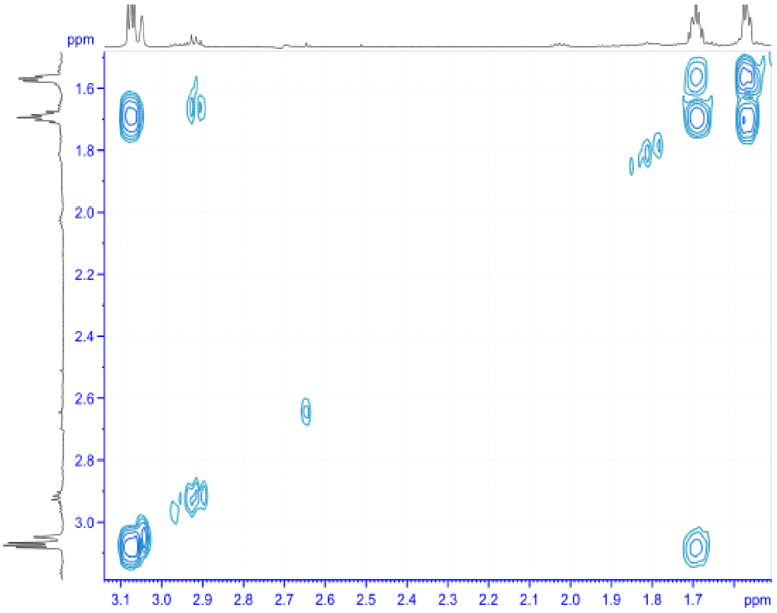

Figure 15S: ^{1}H,^{1}H COSY of piperidine produced in D_2O from cadaverine

Piperidine standard

^{1}H NMR (500 MHz, D_2O) δ (ppm): 1.47 (4-H, q, J = 7.5 Hz), 1.73 (3-H, q, J =7.7 Hz), 1.79 (5-H, q, J = 5.5 Hz), 3.03 (6-H, t, J = 7.7 Hz), 3.17 (2-H, t, J = 5.7 Hz).

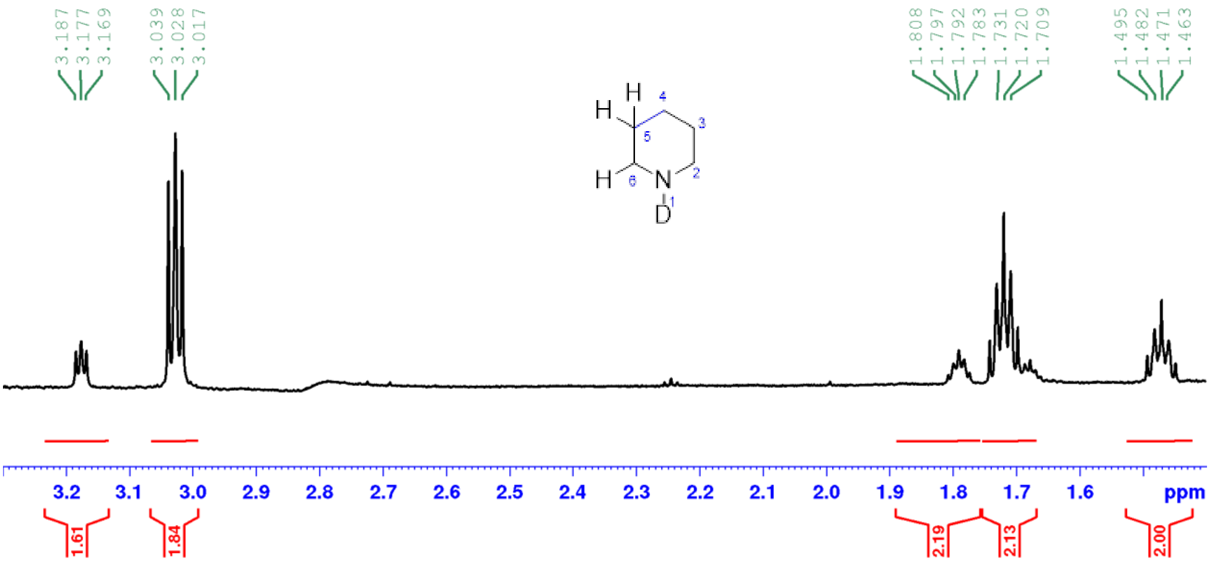

Figure 16S: ^{1}H NMR of piperidine standard

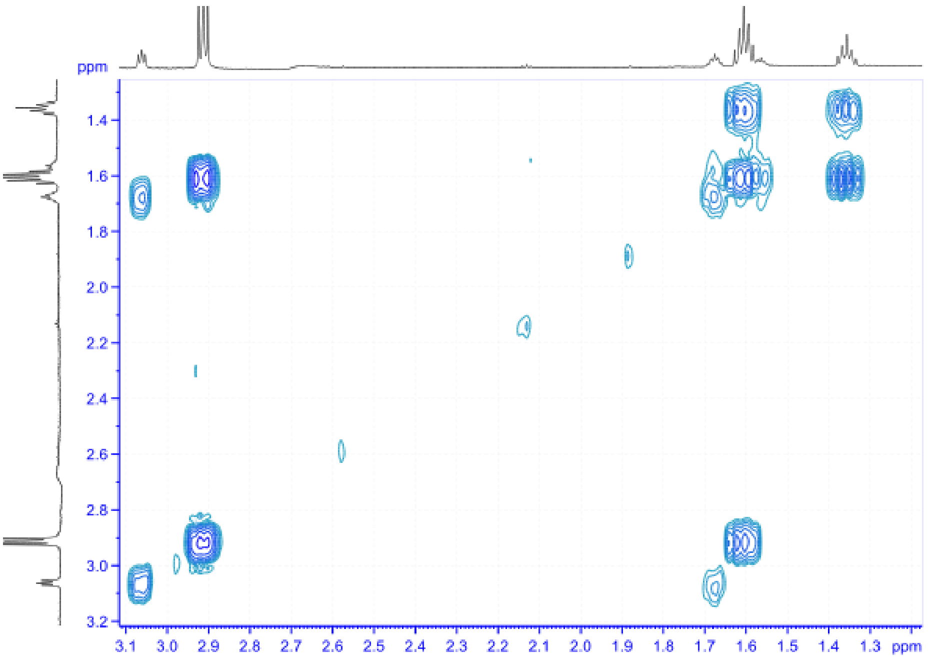

Figure 17S: ^{1}H,^{1}H COSY of commercial standard piperidine

N-methylpiperidine in D_2O

^{1}H NMR (500 MHz, D_2O) δ (ppm): 1.56 (4-H, t, *J* = 5.7, 5.9 Hz), 1.69 (3-H, q, *J* = 6.2, 5.7 Hz), 2.5 (N-methyl, singlet), 3.05 (6-H, singlet), 3.07 (2-H, t, *J* = 5.8 Hz).

The same patterns were observed. Atom 5 was completely labeled with deuterium. The quintet at 1.77 ppm was vanished, in additions the quintet of 4H became triplet. One of H at atom C6 was replaced by deuterium. The triplet at 3.17 ppm was vanished, moreover an additional singlet has appeared at 3.05 indicating the unlabeled H at C6.

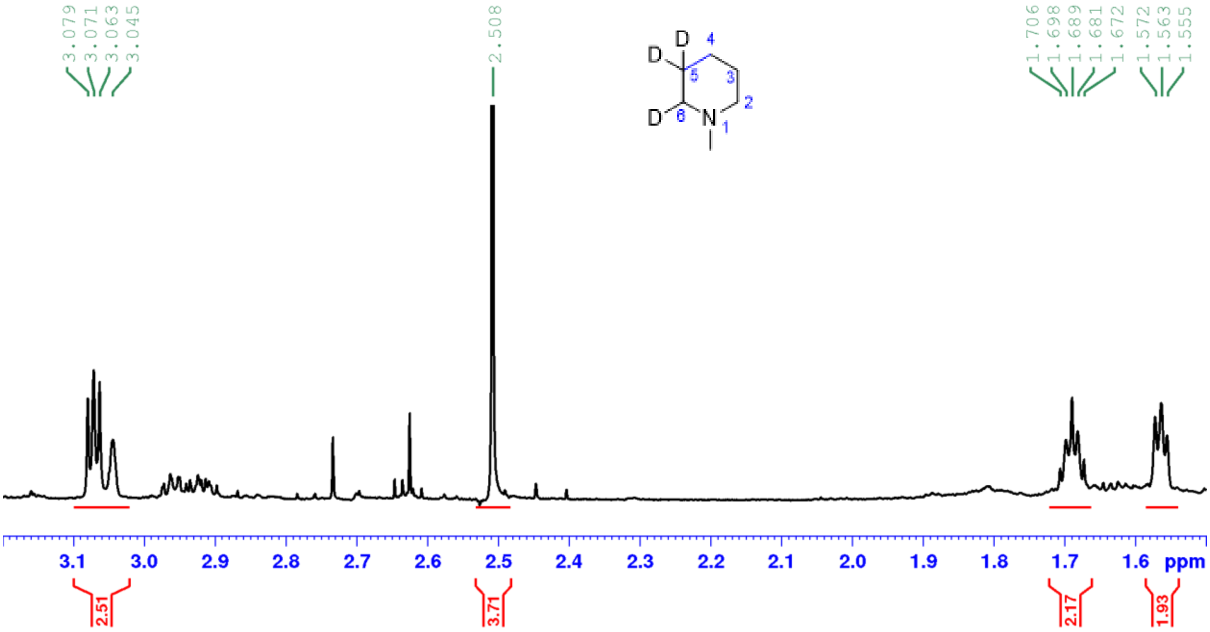

Figure 18S: ^{1}H NMR of *N*-methylpiperidine in D_2O

The NMR of the commercial standard showed the same spectra as the commercial piperidine standard with an additional singlet at 2.5 ppm for *N*-methyl group.

The signals in the NMR spectra for 2-methylpiperidine were too weak to address due to the low yield of the biotransformation.

2.8 Experiments with IRED

In order to determine which atom is isotopically labeled due to SH activity. We tested the isotopic labeling going from imines using only IRED^{NADH} and SH in D_2O. Formate dehydrogenase from *Candida boidinii* (FDH) was used as control. FDH reduces NAD^+ into NADH by transferring the hydride directly from formate to NAD (scheme 1S).[4]

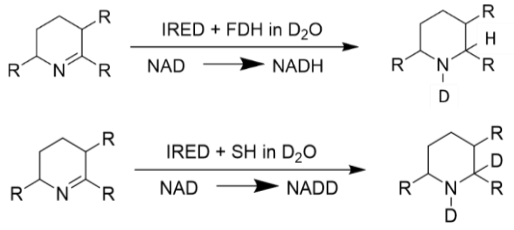

Schema 1S: The reduction of imines into amines catalyzed by IRED^{NADH} in the presence of D_2O with both FDH (above) and SH (below). The isotopic labeling is only possible when SH for cofactor regeneration.

The reaction was performed in Tris-HCl pH 7.5 = pD 8, using 5 mM substrate (Table 4S), 5 mM NAD^+ (neutralized). In case of SH, samples were statured with H_2, and in case of FDH 20 mM formate was added. 3 U of SH / FDH and 15 µM of IRED^{NADH} were added to initiate the reaction. Reaction was carried out in duplicates, overnight at RT and shaking at 80 rpm. Reaction was stopped by adding MeOH (1:1), enzymes were removed by centrifugation. Samples were analyzed using HPLC-MS and NMR.

Table 4S: Substrate used for the experiment and generated product

**Substrate**	**Product**
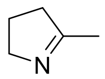 2- methylpyrroline	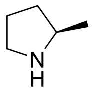 2-methylpyrrolidine
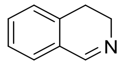 3,4-Dihydroisoquinoline	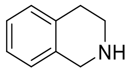 1,2,3,4-Tetrahydroisoquinoline

The mass spectra (Figure 19S) showed as expected a (+1) Dalton in the mass of 1,2,3,4-tetrahydroisoquinoline produced when SH was used as cofactor recycling system, indicating a deuterated product. On the other hand, the setup with FDH did not show any increase in the mass of product and thus no labeling. However, the mass spectra of 2-methylpyrrolidine showed an increase of (+1, +2 and +3) in both systems, this may be caused by the ionization muster of 2-methylpyrrolidine. We observed the same pattern of ionisation when stoichiometric NADH was added and even when the product was acetylated. Therefore, no clear conclusion from the mass spectra in case of methylpyrrolidine can be made. A NMR spectrum was required of both products.

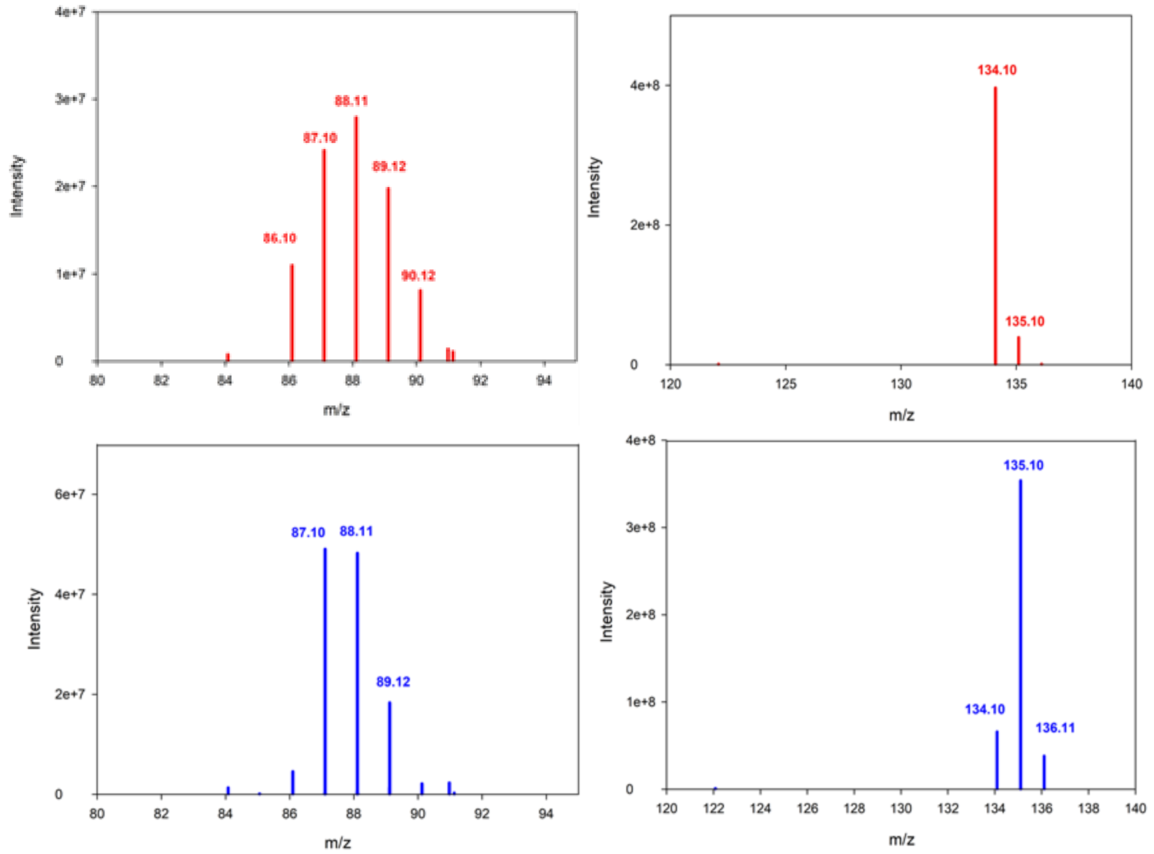

Figure 19S: Mass spectra of 2-methylpyrrolidine produced by the conversion of 2-methypyrroline with FDH as cofactor recycling system (left in red) and SH as recycling system for cofactor (left in blue) as well as of 1,2,3,4 tetrahydroisoquinoline produced by the conversion of 3,4-dihydroisoquinoline with FDH (right in red) and SH as (right in blue).

NMR spectra of 2-methylpyrrolidine (Figure 20S) shows the disappearance of the sextett at 2.87 ppm from the samples, when SH was used for cofactor recycling. The sextet is still present in the samples with FDH and standard - there is a shift of 0.25 ppm thought out the whole spectrum of the standard due to the change in the pH. This indicates a clear labeling of atom Nr.2 with deuterium. Moreover, the doublet of quartet corresponding to atom 3 at 1.05 ppm became a quartet in SH samples. Also the equally doublet of methyl in the standard became dominated by a singlet in SH samples. These all confirm the exchange of H to D at atom C2.

The spectra of 1,2,3,4-tetrahydroisoquinoline (Figure 21S) shows that the high singlet at 3.77 disappeared from the samples with SH and is replaced by a small singlet at 3.5. This indicates an isotopic labeling with deuterium of one of C2 hydrogen atoms. The small singlet may refer to the other remaining atom at C2.

All above mentioned results point out that the isotopic labeling due to SH activity occurs only as expected at the carbon atom of the imine.

2-methylpyrrolidine standard (taking in consideration in the shift of 0.25 ppm): ^{1}H NMR (500 MHz, D_2O) δ (ppm): 0.94 (methyl, d, *J* = 6.6 Hz), 1.097 (3a-H, ddd, *J* = 8.2, 4.1 Hz), 1.59 (3e-H, m), 1.55 (4a-H, m), 2.64 (5a-H, m), 2.78 (5e-H, m), 2.87 (2-H, t, *J* = 7.3, 6.8, 6.4 Hz).

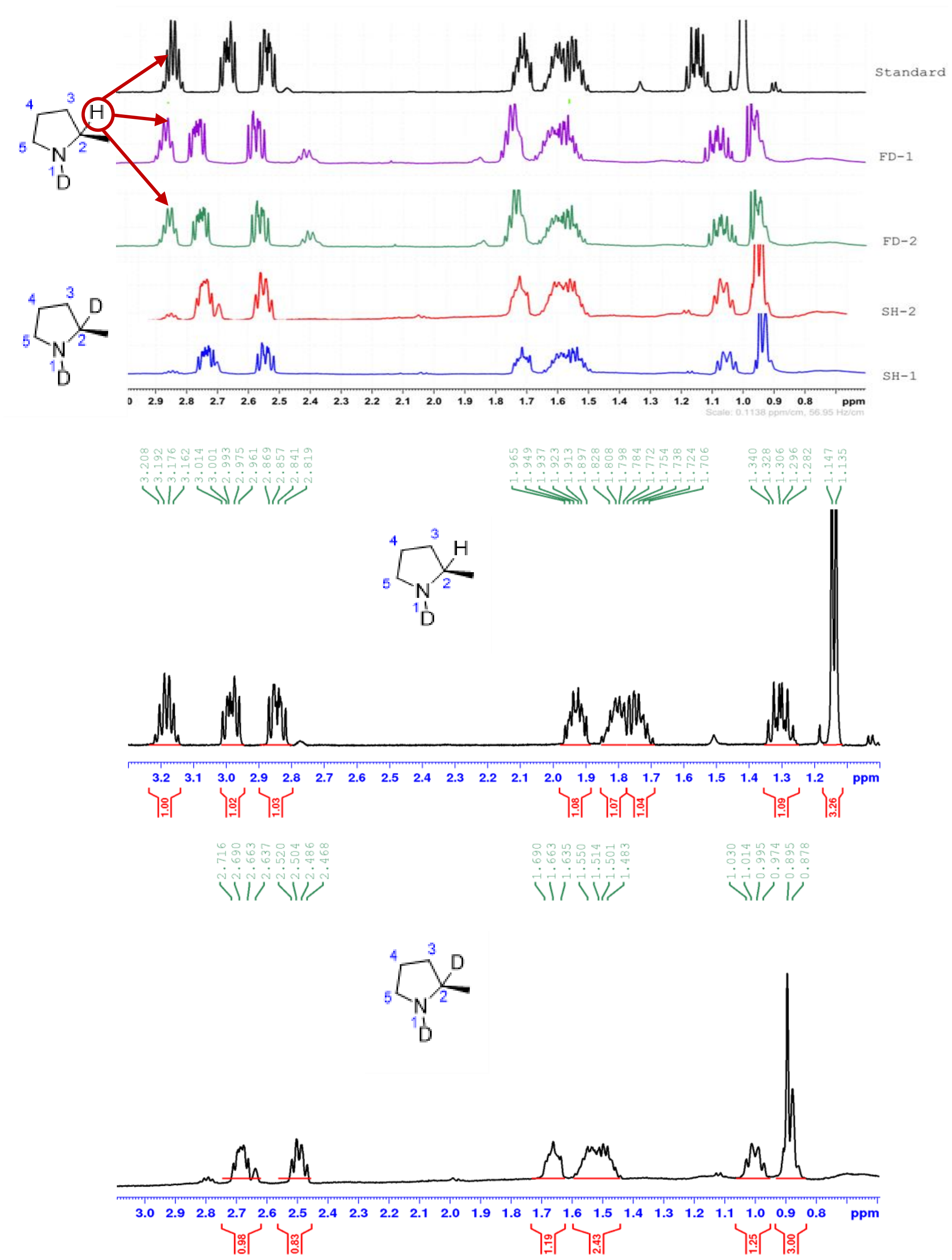

Figure 20S. ^{1}H NMR of 2-methylpyrrolidine in D_2O. Overlay spectra (above). Commercial standard (middle). 2-Methylpyrrolidine produced with SH as cofactor recycling system (below). The standard spectrum shows a shift of 0.25 ppm due to the shift in pH.

1,2,3,4-tetrahydroisoquinoline standard. ^{1}H NMR (500 MHz, D_2O) δ (ppm): 6.9 (aromatic ring), 2.9 (5-H, t, *J* = 6.2, 5.8 Hz), 2.64 (6-H, t, *J* = 6.2, 5.8 Hz), 3.77 (2-H, singlet).

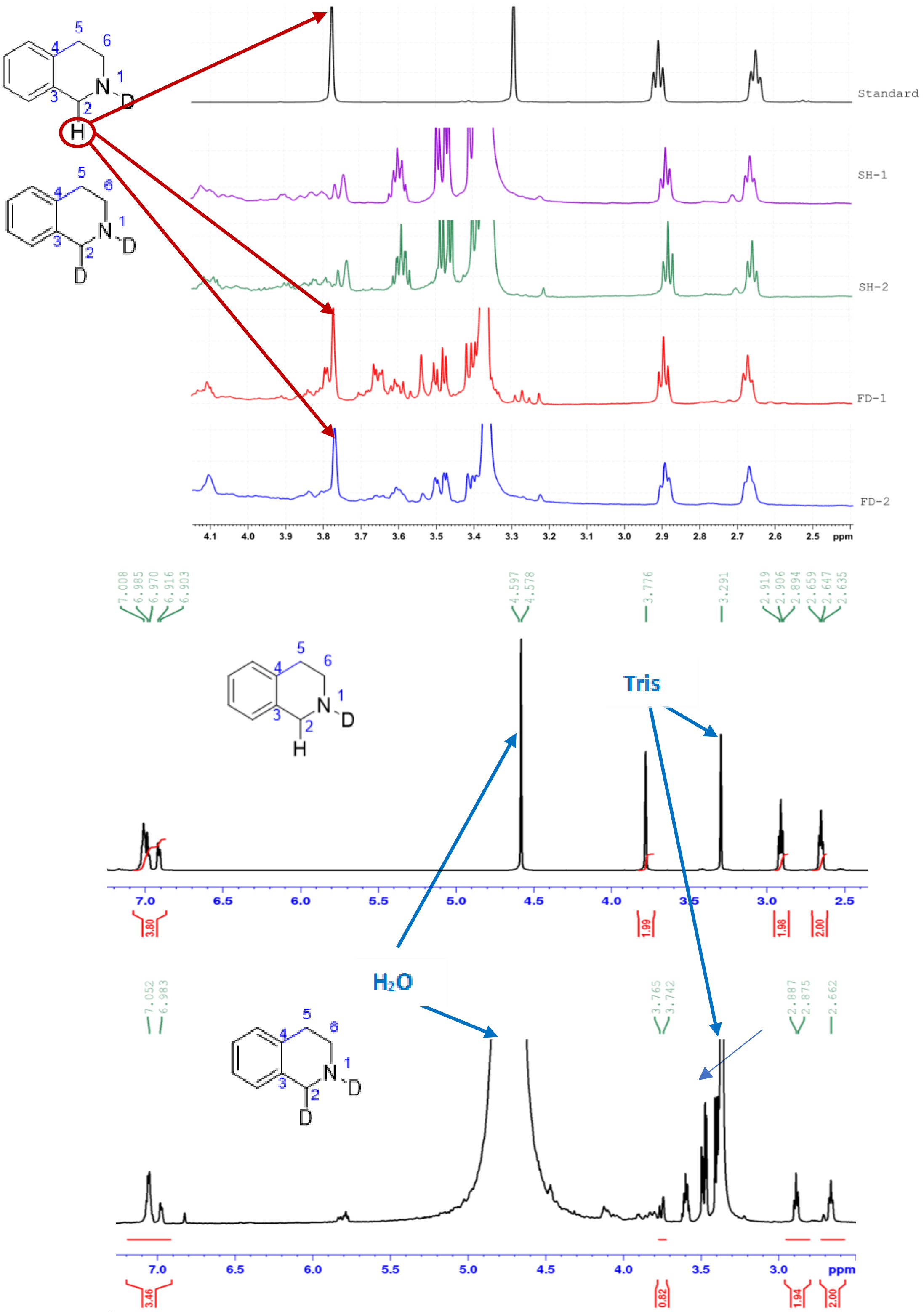

Figure 21S: ^{1}H NMR of 1,2,3,4-tetrahydroisoquinoline in D_2O. Overlay spectra (above). Commercial standard (middle). 1,2,3,4-tetrahydroisoquinoline produced with SH as cofactor recycling system (below).

2.9 Product inhibition of IRED

The product inhibition of IRED^{NADH} was tested at different concentrations using piperidine and 3-methylpiperidine. 5 mM of methylpyrroline was used as substrate with 3 µM IRED^{NADH} and 1mM NADH. The measurement was carried out at RT and pH 7.5. The oxidation of NADH was followed spectrophotometrically at 365 nm (Figure 22S). The enzyme loses 50 % of its activity at 5 mM piperidine and 80 % at 5 mM 3-methylpiperidine.

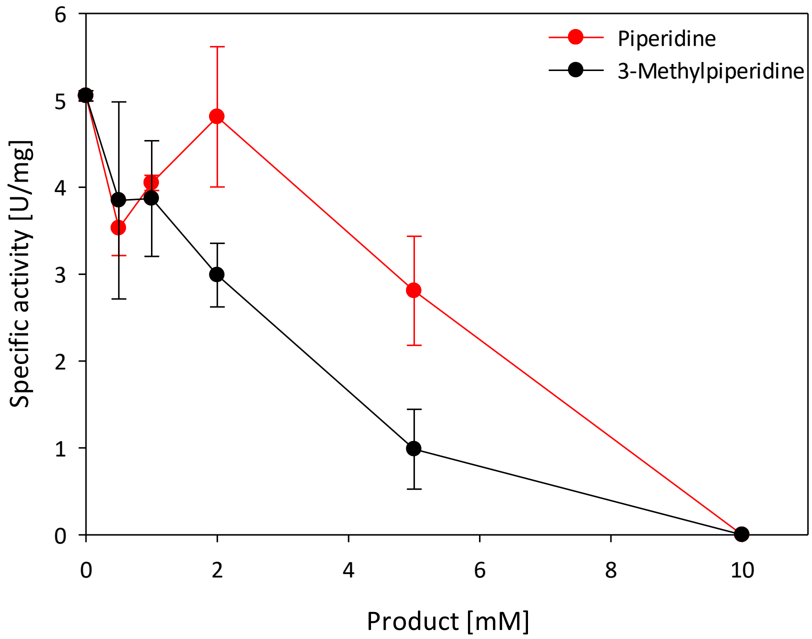

Figure 22S: Product inhibition of IRED^{NADH} at different concentrations of piperidine and 3-methylpiperidine.

2.10 Monitoring product formation

The conversion of diamines to methylated piperidines was analysed over time. The reactions were performed as described in Figure 1, main manuscript.

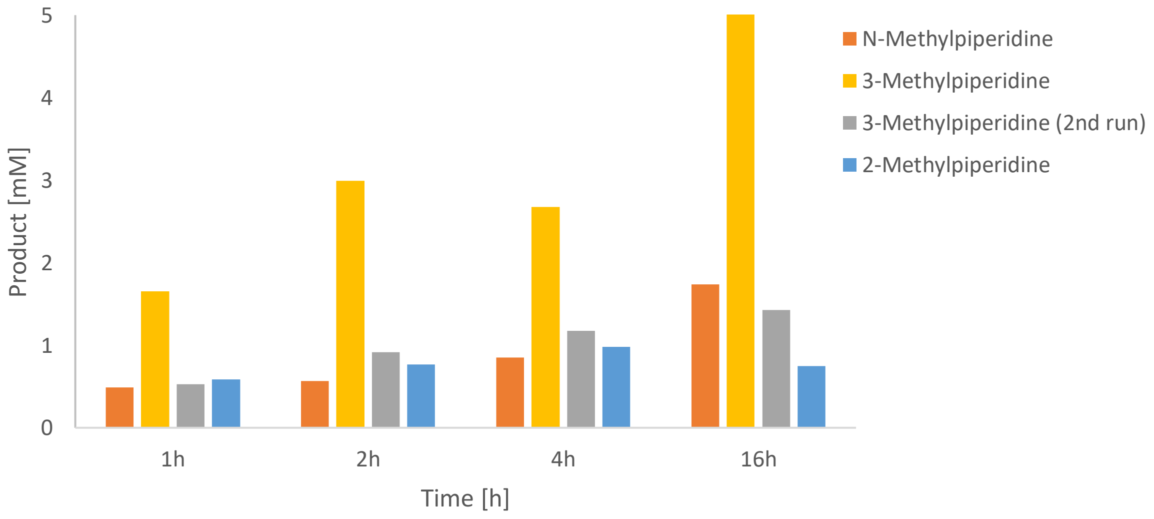

Figure *23S*: Product formation of methylated piperidines over time.

2.11 Calculation for total energy conversion yield

The energy conversion yield was calculated for cadaverine as model substrate. Only molecular hydrogen mediated reaction was considered in the calculations.

X Mol H_2 (produced by electrolysis) → x Mol NADH (produced by SH) → x Mol product (generated by IRED)

The electrolysis was performed for 1 h at 3.5 V and pH 2.7 using pentalandlite/Ni electrodes. After the electrolysis was stopped 2 mM of NAD^+ and 10 mM substrate were added. The reaction was carried out for 16 hours. The electrical current was 50 mA. The cascade produced a total of 33.8 µmol piperidine.

<u>Calculations</u>

$Q = I * t$

$Q = n * z * F$ (F = 96485.3321 C mol^{-1}), z = 2 for H_2

Total electrical charge = 50 mA * 3600 s = 180 As

n = 180 As / 2 * 96485.3321 C mol^{-1}

H_2 = 933 µmol (theoretical)

Conversion = total yield/ total H_2 (theoretical).

= 33.8 / 933

= 0.036

= 3.6 %

*Similar yields were obtained for the other products and for Pt/Pt system.

3 References

[1] A. Al-Shameri, N. Borlinghaus, L. Weinmann, P. Scheller, B. M. Nestl, L. Lauterbach, *Green Chem.* **2019**, *21*,1397-1400.
[2] L. Lauterbach, O. Lenz, *J. Am. Chem. Soc.* **2013**, *135*, 17897-17905.
[3] N. Herr, J. Ratzka, L. Lauterbach, O. Lenz, M. B. Ansorge-Schumacher, *J. Mol. Catal. B: Enzym.* **2013**, *97*, 169-174.
[4] V. O. Popov, V. S. Lamzin, *Biochem J.* **1994**, *301 (Pt 3)*, 625-643.
